# Changes in an enzyme ensemble during catalysis observed by high-resolution XFEL crystallography

**DOI:** 10.1126/sciadv.adk7201

**Published:** 2024-03-27

**Authors:** Nathan Smith, Medhanjali Dasgupta, David C. Wych, Cole Dolamore, Raymond G. Sierra, Stella Lisova, Darya Marchany-Rivera, Aina E. Cohen, Sébastien Boutet, Mark S. Hunter, Christopher Kupitz, Frédéric Poitevin, Frank R. Moss, David W. Mittan-Moreau, Aaron S. Brewster, Nicholas K. Sauter, Iris D. Young, Alexander M. Wolff, Virendra K. Tiwari, Nivesh Kumar, David B. Berkowitz, Ryan G. Hadt, Michael C. Thompson, Alec H. Follmer, Michael E. Wall, Mark A. Wilson

**Affiliations:** ^1^ Department of Biochemistry and Redox Biology Center, University of Nebraska-Lincoln, Lincoln, NE 68588, USA.; ^2^ Computer, Computational, and Statistical Sciences Division, Los Alamos National Laboratory, Los Alamos, NM 875405, USA.; ^3^ Center for Nonlinear Studies, Los Alamos National Laboratory, Los Alamos, NM 87545, USA.; ^4^ Linac Coherent Light Source, SLAC National Accelerator Laboratory, Stanford University, Menlo Park, CA 94025, USA.; ^5^ Stanford Synchrotron Radiation Lightsource, SLAC National Accelerator Laboratory, Stanford University, Menlo Park, CA 94025, USA.; ^6^ Molecular Biophysics and Integrated Bioimaging Division, Lawrence Berkeley National Laboratory, Berkeley, CA 94720, USA.; ^7^ Department of Chemistry and Biochemistry, University of California, Merced, CA 95340, USA.; ^8^ Department of Chemistry, University of Nebraska-Lincoln, Lincoln, NE 68588, USA.; ^9^ Division of Chemistry and Chemical Engineering, California Institute of Technology, Pasadena, CA 91125, USA.; ^10^ Department of Chemistry, University of California-Irvine, Irvine, CA 92697, USA.

## Abstract

Enzymes populate ensembles of structures necessary for catalysis that are difficult to experimentally characterize. We use time-resolved mix-and-inject serial crystallography at an x-ray free electron laser to observe catalysis in a designed mutant isocyanide hydratase (ICH) enzyme that enhances sampling of important minor conformations. The active site exists in a mixture of conformations, and formation of the thioimidate intermediate selects for catalytically competent substates. The influence of cysteine ionization on the ICH ensemble is validated by determining structures of the enzyme at multiple pH values. Large molecular dynamics simulations in crystallo and time-resolved electron density maps show that Asp^17^ ionizes during catalysis and causes conformational changes that propagate across the dimer, permitting water to enter the active site for intermediate hydrolysis. ICH exhibits a tight coupling between ionization of active site residues and catalysis-activated protein motions, exemplifying a mechanism of electrostatic control of enzyme dynamics.

## Teaser

Enzyme catalysis involves complex changes in active site charge and conformation observed using computation and experiment.

## INTRODUCTION

The role of enzyme dynamics in catalysis is one of the most intensively investigated topics in modern biophysics (*1*–*3*). Enzymes, like all proteins, exist in conformational ensembles corresponding to multiple populated minima in their potential energy landscapes (*4*, *5*). Protein dynamics are a combination of motions within these minima and transitions among them. The conformations that compose an ensemble can have intrinsically different catalytic proficiencies, permitting sampling of optimal conformations during catalysis (*6*, *7*), in the laboratory (*8*, *9*), or through evolution (*10*–*12*). Because the catalytic cycle transiently changes the underlying protein energy landscape, enzyme conformational ensembles also change during catalysis (*7*, *13*, *14*). Although equilibrium or near-equilibrium enzyme ensembles are more accessible to study using biophysical approaches, understanding how enzymes facilitate their remarkable rate enhancements ultimately requires directly observing their nonequilibrium behavior during catalysis.

Time-resolved x-ray crystallography can be used to study the nonequilibrium behavior of proteins. Pioneering work using Laue crystallography (*15*–*19*) paved the way for a new generation of time-resolved experiments that use serial x-ray crystallography at x-ray free electron laser (XFEL) (*20*, *21*) or synchrotron (*22*, *23*) sources to follow the responses of proteins to perturbation. By collecting data from thousands of microcrystals, serial crystallography permits crystal manipulation by soaking in substrates (*24*, *25*), activation by light (*26*, *27*), temperature jump (*28*), or releasing caged substrates (*29*, *30*) as well as markedly reducing radiation damage effects (*31*, *32*). Application of time-resolved crystallography and computational methods to several enzymes has revealed that catalysis can activate enzyme motions not apparent in the absence of substrate (*33*, *34*). However, interpretation of the difference electron density maps produced by time-resolved crystallography experiments can be challenging. Minor conformations populated stochastically rather than in concert during catalysis are especially difficult to identify and model, although recent advances in calculating weighted electron density maps help resolve these conformations (*35*, *36*). Because these minor conformations can play important roles in catalysis (*37*) and in contributing to protein entropy (*38*), it is imperative to develop strategies to enrich them to study important regions of the reaction coordinate.

**Fig. 1.**
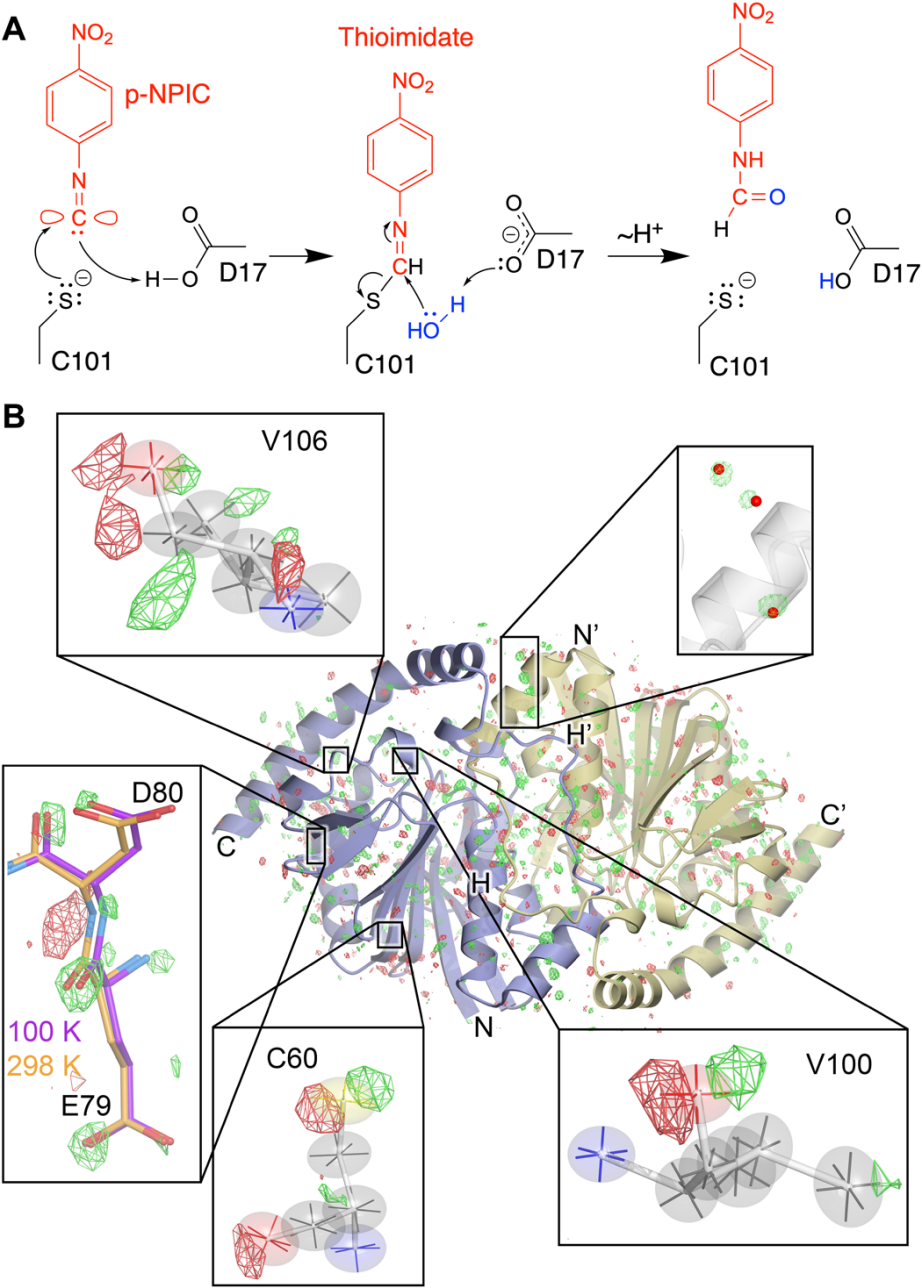
ICH mechanism and evidence for temperature-dependent modes of intrinsic enzyme flexibility. (**A**) Postulated ICH mechanism is shown for the para-nitrophenyl isocyanide (p-NPIC) substrate used in this study. The isocyanide is shown in its carbenoid form, consistent with the electrophilic character needed for attack by the catalytic Cys^101^ thiolate nucleophile in ICH. Formation of the thioimidate intermediate is postulated to be facilitated by the general acid Asp^17^, which protonates the C1 atom and then later acts as a general base to activate water (blue) for thioimidate hydrolysis. (**B**) Temperature-dependent changes in the ICH dimer. The *F*_o_(274 K synchrotron)-*F*_o_(298 K XFEL) isomorphous difference electron density map is contoured at 3σ, with positive features shown in green and negative features in red. Insets show regions of special interest, including the alignment of difference electron density features along the principal axes of anisotropic ADPs in Cys^60^, Val^100^, and Val^106^. *F*_o_(274 K synchrotron)-*F*_o_(298 K XFEL) peaks near ordered waters (red spheres, top) indicate higher occupancy of these waters at 274 K than 298 K. For residues 79 and 80, the difference electron density indicates displacements that agree with the structural differences observed in a 100 K crystal structure (purple bonds) compared to the 298 K XFEL structure (gold bonds).

Our enzyme of interest is isocyanide hydratase (ICH), which catalyzes the irreversible hydration of isocyanides to N-formamides via formation of a cysteine-derived thioimidate intermediate (Fig. 1A) (*33*, *39*–*41*). Isocyanides (also called isonitriles) have a triple bonded nitrogen-carbon (R-N^+^≡C^−^) functional group in resonance with a double-bonded electrophilic carbenoid species (R-N═C). These compounds often have antibiotic and chalkophore (copper-binding) activities (*42*–*46*). ICHs are commonly found in pseudomonad bacteria and soil-dwelling fungi and likely serve a defensive role against isocyanide natural products produced by competing microbes (*40*, *47*). In addition to its intrinsic biochemical interest, ICH is a useful model system for catalysis-activated enzyme dynamics. Prior XFEL mix-and-inject serial crystallography (MISC) studies of *Pseudomonas* ICH showed that formation of the thioimidate intermediate causes the enzyme to populate a different conformational ensemble during catalysis (*33*). In wild-type ICH, covalent modification of the active site cysteine thiolate led to distributed changes in the ICH conformational ensemble across the dimer, especially for helix H (residues 152 to 166) near the active site in protomer A (*33*) (fig. S1). However, the isomorphous (*F*_o_-*F*_o_) difference electron density around mobile regions of the enzyme was difficult to interpret on its own, and structures of ICH with an oxidized Cys^101^ nucleophile that enriched these conformations were needed to confirm a structural model where helix H moved upon intermediate formation owing to a loss of negative charge on the Cys^101^ thiolate. Two mutations (G150T and G150A) that reduced ICH catalytic rate approximately sixfold were also engineered for that study. The 277 K crystal structure of G150T ICH showed that helix H is shifted, similar to a minor conformation sampled by wild-type ICH when Cys^101^ is modified by formation of the catalytic intermediate (*33*). Because G150T stably adopts a state that is transiently populated by the wild-type enzyme, it is a useful tool for studying how ICH conformational ensembles change during the catalytic cycle.

Here, we use MISC (*24*, *25*) at the Linac Coherent Light Source (LCLS) XFEL to observe how catalysis affects the conformational ensemble of G150T ICH. The high resolution (1.3 Å) of the G150T ICH XFEL MISC data permits building detailed structural models for the resting enzyme and the catalytic intermediate as well as refinement of anisotropic atomic displacement parameters (ADPs) for both structures. Changes in the protonation of active site residues during catalysis modulates G150T ICH conformational dynamics, and this mutant provides a clearer view of a key step in ICH catalysis than was possible with the wild-type enzyme. The relevance of these results to time-resolved studies of other enzymes is discussed.

## RESULTS

### XFEL and synchrotron crystal structures of G150T ICH have differences that lie along intrinsic modes of enzyme flexibility

We obtained a 1.3-Å resolution XFEL structure of resting G150T ICH at 298 K using 20- to 30-μm microcrystals delivered via a microfluidic electrokinetic sample holder (MESH) injector at the Macromolecular Femtosecond Crystallography (MFX) beamline at LCLS (see Methods). The refined XFEL structure of G150T ICH superimposes nearly exactly [0.14-Å all-atom root mean square deviation (RMSD)] with previous 1.1- to 1.2-Å resolution 274 K synchrotron structures of this mutant protein (*48*), as expected. However, there are surprising differences between the XFEL and synchrotron structures evident in *F*_o_(synchrotron)-*F*_o_(XFEL) isomorphous difference electron density maps, which contain over 300 peaks at 3.2σ (0.2 e^−^/Å^3^) or higher within 5 Å of the protein and ordered solvent model (Fig. 1B). By comparison, a *F*_o_-*F*_o_ map of two replicate synchrotron G150T datasets has no features above 0.1 e^−^/Å^3^, establishing that the *F*_o_(synchrotron)-*F*_o_(XFEL) peaks are genuine signal. Several of the largest positive difference peaks (5σ to 6σ) are near ordered solvent and indicate higher occupancy of these waters in the 274 K synchrotron dataset (Fig. 1B). Other peaks are near residues sampling multiple conformations (e.g. Cys^67^ and Cys^101^) and suggest higher occupancies for minor conformers in the 298 K XFEL dataset (fig. S2). In cases where positive and negative *F*_o_(synchrotron)-*F*_o_(XFEL) peaks are paired near an atom, these features lie roughly along a principal axis of the anisotropic ADP ellipsoid for well-ordered and electron-dense atoms, such as peptide oxygen and sulfur atoms in Val^100^, Val^106^, and Cys^60^ (Fig. 1B). The correspondence between these difference map features and the directional preferences of the anisotropic ADPs suggests that small atomic displacements between the synchrotron and XFEL datasets occur along intrinsically preferred directions of atomic motion in the protein.

The movement of atoms along “softer” internal degrees of freedom in the enzyme is further supported by comparison of a cryogenic (100 K) structure with the 274 K synchrotron and 298 K XFEL structures of G150T ICH. The cryogenic structure superimposes on the XFEL structure of G150T with all-atom RMSD of 0.45 Å (Cα RMSD = 0.21 Å), and many *F*_o_(synchrotron)-*F*_o_(XFEL) map peaks lie in the direction of displacements between the cryogenic and XFEL structures (Fig. 1B). We observe these difference peaks in *F*_o_(synchrotron)-*F*_o_(XFEL) maps calculated from three independent 274 K G150T ICH synchrotron datasets (*48*), demonstrating reproducibility (fig. S3). A plausible explanation for these difference electron density features is the 24°C difference in temperature between the synchrotron and XFEL datasets, which can be presumed to shift conformational equilibria. Minor radiation damage to the synchrotron dataset or differences in the sample environment during synchrotron and XFEL data collection cannot be ruled out as potential contributing factors but are unlikely to be dominant effects (*49*, *50*). Substantial differences between room temperature and cryogenic crystal structures are well documented (*51*–*55*), and protein conformational ensembles can respond in complex ways as temperature is increased (*56*). Consistent with these previous studies, our results indicate that even moderate changes in temperature that are well above the “glass transition” at ~180 K leave a clear imprint on protein conformational heterogeneity in atomic resolution isomorphous difference electron density maps. Moreover, our observation that these features correlate with preferred directions of atomic motion inferred from anisotropic ADPs supports the physical validity of the directional information in the anisotropic ADPs.

### The resting conformational ensemble of G150T ICH is sampled by wild-type ICH during active site protonation and catalysis

**Fig. 2.**
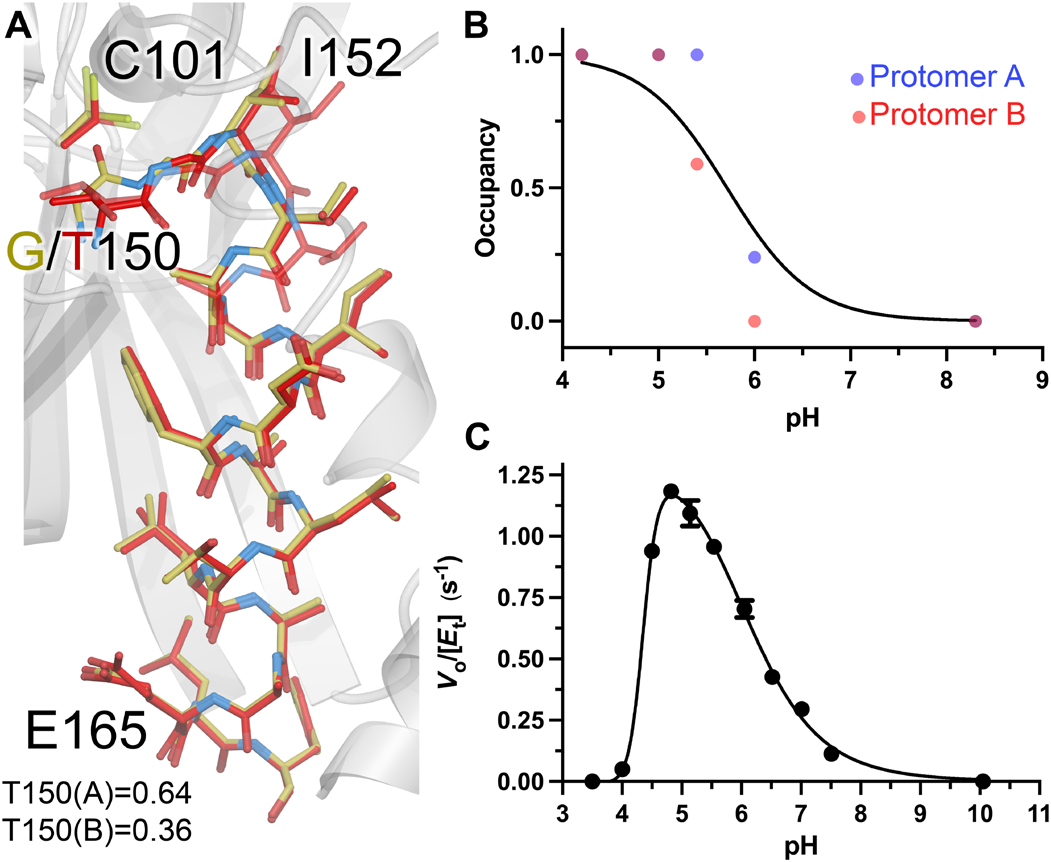
G150T ICH predominantly populates conformations that are sampled by wild-type ICH upon Cys^101^ charge neutralization. (**A**) Close structural agreement between the shifted conformation of helix H in resting G150T at pH 8.8 (red) and wild-type ICH at pH 4.2 (gold). Alternate conformations are shown as semi-transparent bonds, and occupancies are shown at the bottom. (**B**) Helix H conformational changes owing to Cys^101^ protonation. The refined occupancies of the shifted (i.e., G150T-like) conformation of helix H in crystal structures of wild-type ICH are plotted against pH. ICH crystallizes with two molecules in the ASU, with occupancies of helix H for protomer A shown as blue points and protomer B as red. The data are fitted to the Henderson-Hasselbalch equation (black; see Methods) with an apparent p*K*_a_ of 5.7. (**C**) pH versus rate profile for wild-type ICH. Data were measured in triplicate, with error bars showing SEM and fitted using a dose-response curve with a rising inflection point at 4.4, maximum at 4.8, and falling inflection point of 6.1.

The G150T ICH structure overlaps with features in the isomorphous difference electron density maps calculated using previously collected wild-type ICH XFEL data before and after formation of the thioimidate intermediate (fig. S4), demonstrating that G150T ICH natively occupies a state that wild-type ICH samples transiently upon catalytic intermediate formation. In previous work, we proposed that the thioimidate intermediate neutralizes the negative charge of Cys^101^ thiolate, modulating a network of H-bonds that facilitate conformational changes across the ICH dimer (*33*). By contrast, G150T ICH samples this conformational ensemble at rest because of steric effects of the G→T substitution at residue 150. Because thioimidate formation transiently adds a bulky group to Cys^101^ during catalysis, it seemed plausible that some previously observed changes in wild-type ICH conformational ensemble might be due to steric effects rather than changes in Cys^101^ charge. To test the hypothesis that neutralizing the negative charge on the C101 thiolate is sufficient to cause wild-type ICH to sample G150T-like conformations, we determined synchrotron crystal structures at 100 K of wild-type ICH at various pH values in the range of 4.2 to 8.3. We hypothesized that the Cys^101^ thiolate would become protonated at lower pH values, recapitulating the loss of the negative charge on the Cys^101^ Sγ atom caused by covalent bond formation during catalysis. Although it is possible that other ionizable groups could contribute to the change in the ICH conformational ensemble, previous results suggest that Cys^101^ is of central importance (*33*, *39*). Consistent with these predictions, pH values below 6.0 cause a shift in wild-type ICH helix H to a position that is nearly identical (Cα RMSD = 0.18 to 0.19 Å) to the resting G150T ICH helix H from residues 152 to 164 (Fig. 2A). Wild-type ICH structures at pH 5.4 and 6.0 contain both strained and shifted helix H conformations, while structures at pH 5.0 and 4.2 show only the shifted helix conformation, suggesting that the Cys^101^ p*K*_a_ (where *K*_a_ is the acid dissociation constant) value is in the range of 5 to 6 (Fig. 2B and figs. S5 and S6). This inference is supported by the pH dependence of ICH activity, which has a maximum at pH 4.8 (Fig. 2C). This value is within 1 unit of the p*K*_a_ values of homologous cysteine residues in related DJ-1 superfamily proteins (*57*–*59*). Therefore, using pH as a tool to perturb ICH conformational ensembles shows that helical mobility in ICH is closely correlated with Cys^101^ charge state and that the G150T mutant recapitulates the wild-type ICH conformational ensemble when Cys^101^ is protonated or covalently modified. Although the G150T mutant, wild-type ICH at low pH, a previously characterized C101A mutant (*39*), and Cys^101^-SOH oxidized ICH (*33*) all sample similar ensembles with a shifted helix H, only G150T is catalytically active. The activity of G150T ICH is essential for this work because it allows us to characterize changes in its conformational ensemble during catalysis and to relate these to infrequently sampled conformations of the wild-type enzyme.

### Substrate selects catalytically competent G150T ICH active site conformations

**Fig. 3.**
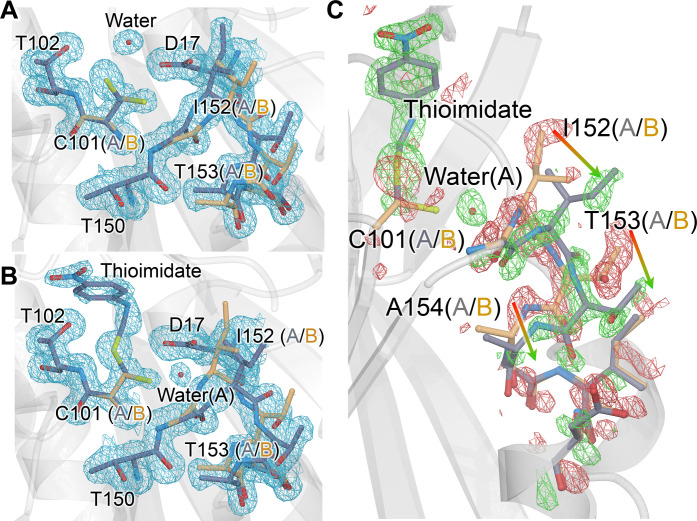
Formation of thioimidate intermediate selects catalytically competent active site conformations and redistributes the G150T ICH ensemble. (**A**) The 1.3-Å resolution 2m*F*_o_-D*F*_c_ XFEL electron density contoured at 0.9σ (blue) for the free G150T ICH at 298 K. Alternate conformations for active site residues are blue (conformer A) and gold (conformer B). (**B**) The 1.3-Å resolution 2m*F*_o_-D*F*_c_ XFEL electron density contoured at 0.9σ (blue) for the thioimidate intermediate observed 30 s after mixing microcrystals with p-NPIC substrate in a mix-and-inject serial crystallography (MISC) experiment. Only conformer A of Cys^101^ reacts with the intermediate, which redistributes the conformational disorder in the I152 loop. (**C**) Redistribution of the populations of the active site ensemble upon formation of the thioimidate intermediate. The *F*_o_(30 s)-*F*_o_(0 s) isomorphous difference electron density map is contoured at 3σ (green, positive/red, negative). Arrows show the direction of population shift, from red (loss of population) to green (gain of population). A partially occupied water (red sphere) enters the active site upon formation of the intermediate.

To observe catalysis in G150T ICH microcrystals, we performed a MISC experiment by mixing the substrate para-nitrophenyl isocyanide (p-NPIC) with G150T ICH microcrystals using the coMESH injector and determined a 1.3-Å resolution XFEL crystal structure at 298 K. Like wild-type ICH (*33*), we found that larger (>100 μm) G150T ICH crystals are damaged by the introduction of substrate, necessitating the serial microcrystallography approach taken here. The resting structure of G150T has pronounced conformational disorder at the active site, providing an opportunity to observe how this ensemble of conformations responds to catalysis. In the resting state of G150T ICH, the Cys^101^ nucleophile populates two major conformations, one corresponding to the conformation observed in wild-type ICH and another that would conflict with Ile^152^ in the resting wild-type ICH conformation. The shifted helix H in G150T ICH opens a pocket that permits this conformational heterogeneity at Cys^101^ (Fig. 3A). After 30 s of mixing with substrate, electron density maps show formation of the covalent thioimidate intermediate at Cys^101^ in G150T ICH (Fig. 3B and fig. S7). The substrate selects the native-like, catalytically competent Cys^101^ conformation exclusively, with no evidence of thioimidate formation on the second Cys^101^ conformation (Fig. 3B). The absence of thioimidate electron density on the second Cys^101^ conformation is most consistent with a model where the alternate conformation of Cys^101^ is catalytically inert and that Cys^101^ stops sampling the alternate conformation once the catalytically competent Cys^101^ conformation reacts with p-NPIC to form the thioimidate. We note, however, that we cannot exclude a model whereby reaction of the substrate with the minor Cys^101^ conformation results in it moving to exclusively populate the major conformation. The refined occupancy of the thioimidate is 0.53, which is less than the 0.85 occupancy of the Cys^101^ conformer to which it is bonded, indicating partial formation of thioimidate after 30 s. Therefore, the catalytically competent Cys^101^ conformation is 62% modified to the intermediate in this structure. G150T ICH provides a clear example of catalytic modification of the cysteine nucleophile causing changes to active site conformational dynamics and affords a high-resolution view of combined conformational and chemical heterogeneity in an enzyme active site.

Formation of the intermediate results in strong *F*_o_(30 s)-*F*_o_(0 s) isomorphous difference electron density extending from the active site to surrounding residues (Fig. 3C). The loop comprising residues 151 to 154 samples two conformations in G150T (Fig. 3C), with the one nearer to Cys^101^ (conformer A in the free enzyme, conformer B in the thioimidate) being more populated when the enzyme is at rest. This will be called the closed conformer. The *F*_o_-*F*_o_ difference electron density indicates that the populations of the two loop conformations have been redistributed upon intermediate formation, although their structures have not changed (Fig. 3C). The refined occupancy of the closed conformer is reduced from 0.64 in the resting enzyme to 0.39 in the G150T-thiomidiate structure, representing an inversion in the population distribution of these two conformers compared to the free enzyme. However, because the occupancy of the thioimidate is only 0.53, it is likely that a fully occupied catalytic intermediate would result in an even larger reduction in the occupancy of the closed conformer. From a thermodynamic perspective, these two conformers represent local energy minima on the energy landscape of possible G150T ICH configurations, with their relative populations determined by the Boltzmann distribution. The observed change in occupancies between the resting and thioimidate G150T ICH structures shows that thioimidate formation changes the underlying energy landscape such that the relative difference free energy (∆*G*) has increased markedly to favor the open conformer. The redistribution of the populations of these two loop conformations also changes the locations of ordered waters around residues 152 to 154 in helix H and in the surrounding IJ linker region from the other protomer (fig. S8), demonstrating correlated protein-solvent conformational reorganization. This mobile loop contains Ile^152^, whose backbone amide donates an H-bond to the Cys^101^ thiolate and is a severe Ramachandran outlier in the wild-type enzyme (fig. S9A). If that H-bond is weakened, Ile^152^ samples more favorable backbone torsion angles and facilitates the helix shift observed in the wild-type enzyme at low pH (see above) or during catalysis (*33*). In G150T ICH, Ile^152^ is in Ramachandran-permitted regions in both loop conformers (fig. S9B). Therefore, this additional level of loop-helix disorder in G150T ICH was unexpected as it does not appear to be coupled to backbone torsional strain.

### Formation of the catalytic intermediate results in changes to G150T ICH that span active sites in the dimer

**Fig. 4.**
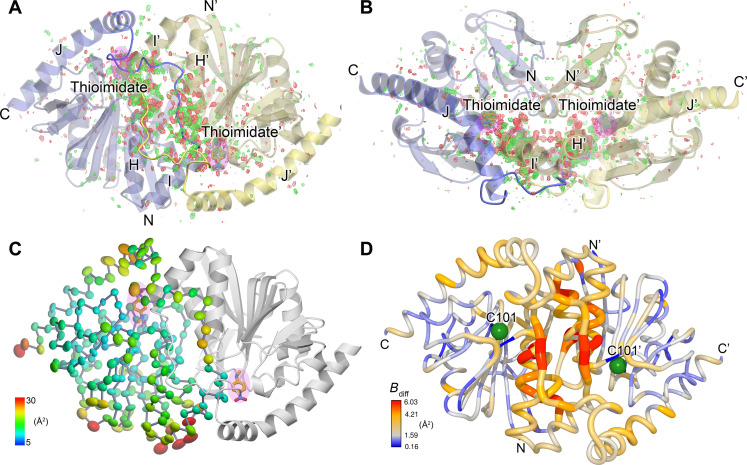
Catalysis causes global changes to G150T ICH dimer conformational ensemble. (**A** and **B**) Formation of the thioimidate intermediate (labeled; pink highlight) causes changes that propagate across the dimer interface. Two views of the G150T ICH dimer related by a 90° rotation about the horizontal axis are shown. The *F*_o_(30 s)-*F*_o_(0 s) isomorphous difference electron density map is contoured at 3σ (green, positive/red, negative). The linker connecting helices I and J is shown in darker colors and mediates inter-protomer dynamics that connect the active sites. (**C**) Anisotropic ADP ellipsoids for Cα atoms in the thioimidate intermediate are shown at the 85% probability level and are colored by magnitude, from blue (5 Å^2^) to red (30 Å^2^). The thioimidate intermediate (labeled; pink highlight) is near areas of elevated mobility. (**D**) Formation of the thioimidate intermediate increases mobility in several areas of G150T ICH including the IJ linker and helix H. Difference ADP values between the thioimidate structure and the free enzyme (*B*_diff_ = *B*_30 s_ − *B*_0 s_) are shown, with scale indicated at the lower left. Areas with higher *B*_diff_ values coincide with peaks in the *F*_o_(30 s)-*F*_o_(0 s) isomorphous difference electron density map [(A) and (B)].

Catalysis-activated motions in ICH extend across the dimer interface, spanning both protomers of the ICH dimer. *F*_o_(30 s)-*F*_o_(0 s) isomorphous difference electron density features are distributed throughout the ICH dimer in an anisotropic manner, with most peaks being clustered near the helix H and stretch of residues between helices I and J (IJ linkers) that span the two active sites (Fig. 4, A and B). We note that G150T ICH crystallizes in space group C2 with one protomer in the asymmetric unit (ASU), meaning the symmetry of these features is a consequence of crystallographic symmetry. However, asymmetry in ICH dynamics typically results in the full dimer being contained in the ASU, as seen in wild-type ICH (*33*). We propose that the fully shifted helix H in G150T ICH causes a symmetrization of ICH protomer dynamics that is obeyed during catalysis in the crystal.

Prior analysis of correlated alternate conformations in wild-type ICH indicated that motions in helix H in one protomer are communicated to the other protomer via the proline-rich IJ linker (*33*). The *F*_o_(30 s)-*F*_o_(0 s) isomorphous difference electron density for G150T shows that even after helix H is shifted, these residues remain a key pathway for dimer-spanning correlated motions. Because each IJ linker region makes contacts with both helices H and H′ in the two protomers in the ICH dimer, displacements in the linker provide a dimer-spanning path between the two active sites (Fig. 4, A and B). The IJ linker is a distinctive feature of ICH homologs and distinguishes them from other close structural relatives in the DJ-1 superfamily (*39*), suggesting that other ICH homologs may exhibit similar IJ linker–mediated dynamical pathways through the ICH dimer.

ADPs provide another measure of atomic motions in crystals, and the 1.3-Å resolution of these data allows for the refinement of anisotropic ADPs that contain information about directional preferences in atomic motion. Despite their potentially high information content, comparing ADPs between multiple crystal structures is complicated by idiosyncratic differences between the crystals used to collect the multiple datasets, leading to mismatches in baseline ADP values and misattribution of crystalline disorder at other length scales to individual atomic motions (*60*). These limitations are partially ameliorated by serial crystallography because a single dataset comprises diffraction images from many crystals, diminishing the influence of individual crystal idiosyncrasies on the refined ADPs. The ADPs in the active site, helix H, and IJ linker regions of the G150T ICH thioimidate intermediate are higher than in the resting enzyme, and these differences correspond closely to the spatial distribution of *F*_o_(30 s)-*F*_o_(0 s) difference map features (Fig. 4) as quantified using integration of the absolute difference density above a noise threshold (IADDAT) (fig. S10, A and B) (*61*). These differences are localized to these regions of the dimer, with similar ADPs in other portions of the protein (Fig. 4D and fig. S10C). Despite the differences in anisotropic ADP magnitudes between the enzyme at rest and during catalysis, there are no major changes in the directionality of the anisotropic ADPs upon thioimidate formation as determined via visual inspection and Rosenfield rigid body analysis (fig. S11) (*62*). Therefore, formation of the thioimidate intermediate changes the magnitudes but not overall directional preferences of atomic displacements in the active site, helix H, and IJ linker regions of ICH.

### Water entry into active site depends on dynamics activated by intermediate formation

**Fig. 5.**
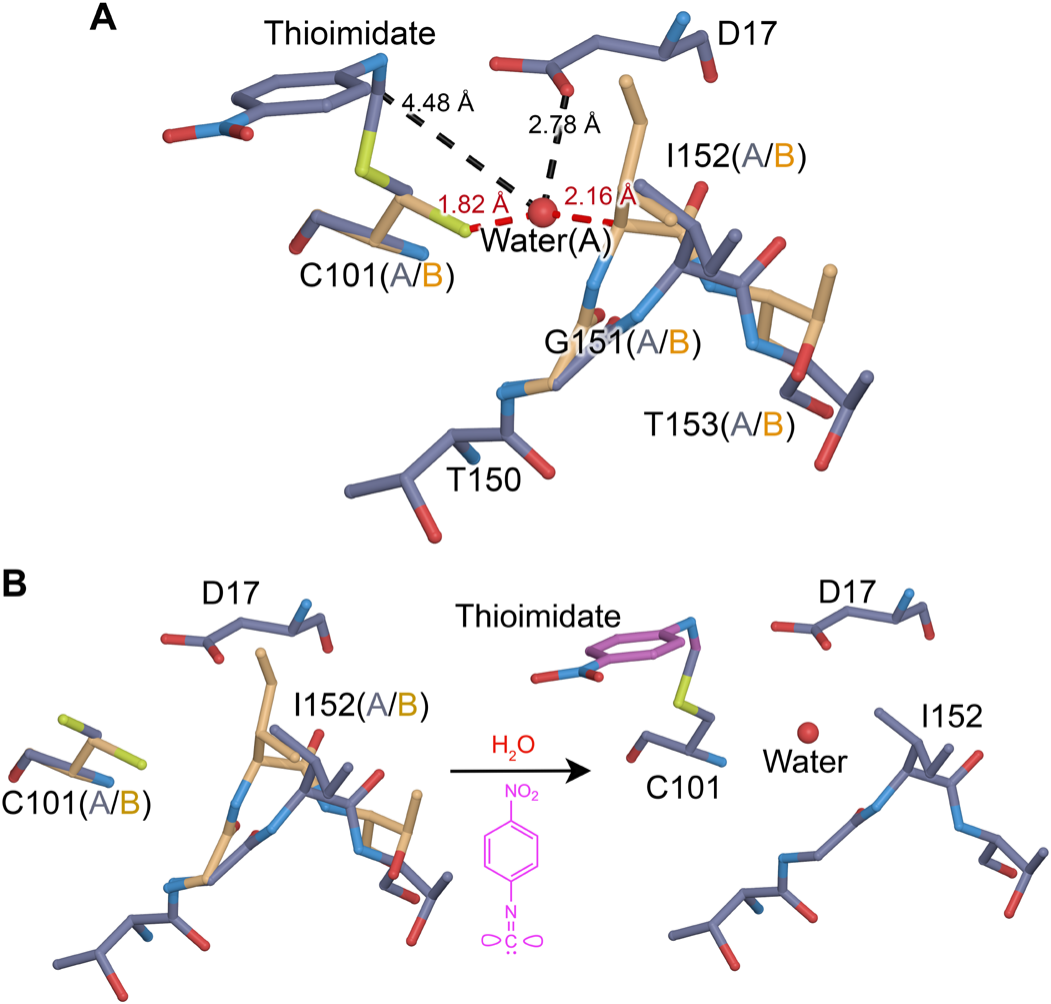
Thioimidate formation and conformational dynamics facilitate water entry into the active site. (**A**) Interactions between the alternate conformations of active site residues and water require correlated motions to avoid steric clashes. The “A” alternate conformation is in slate gray bonds, and the “B” alternate conformation is in gold. Interactions between the partially occupied water (labeled red sphere) and surrounding residues are shown with dotted lines, with distances in angstroms. Red lines indicate steric clashes between the water and residues in the “B” conformation that are avoided by correlated sampling of the nonconflicting alternate “A” conformations. (**B**) Changes in the conformational heterogeneity of the active site upon thioimidate formation and water entry. Reaction of Cys^101^ with p-NPIC (purple) forms a thioimidate intermediate (purple) and allows water to enter the active site. If both the thioimidate and water are fully occupied, conformational heterogeneity in the active site is reduced to avoid steric clashes.

The ICH mechanism requires hydrolysis of the thioimidate intermediate, although water has not been observed in a catalytically plausible location in previous XFEL electron density maps of the wild-type enzyme during catalysis. By contrast, the G150T ICH thioimidate electron density has a peak consistent with water between Cys^101^ and Ile^152^ and 4.3 Å away from the thioimidate carbon atom in the scissile C-S bond. This water enters the active site when the thioimidate intermediate forms, as shown by a 5.1σ positive peak in the *F*_o_(30 s)-*F*_o_(0 s) isomorphous difference electron density map and the absence of any peak in 2m*F*_o_-D*F*_c_ maps of the resting enzyme (Fig. 3). The water is only partially occupied (refined occupancy = 0.64) because its entry into the active site requires motion of the 151–154 loop to relieve a steric conflict between the water and Ile^152^ Cα and Hα atoms (Fig. 5A). Moreover, this water can enter this site only when Cys^101^ is in its dominant, catalytically competent conformation because of a steric clash with the minor Cys^101^ conformation (Fig. 5A). Therefore, correlated disorder at multiple locations in the G150T ICH active site gates access of the water molecule to this binding pocket upon formation of the catalytic intermediate (Fig. 5B). This water molecule is not optimally positioned to initiate catalysis, being both too far from the C-S bond and not optimally positioned for an in-line attack and displacement of the Cys^101^ S atom (Fig. 5A). Therefore, it is possible that it is not the hydrolytic water. However, it is the closest water to the intermediate in the active site and enters the active site upon intermediate formation, suggesting that it is catalytically relevant. The G150T mutant structure provides a clearer view of this state than would be possible in the wild-type enzyme because the shifted helix H conformation required for water entry is transiently sampled with low occupancy once the intermediate forms in wild-type ICH but is the dominant conformation of G150T ICH.

### Crystalline molecular dynamics simulations show that active site dynamics are coupled to Asp^17^ deprotonation

Asp^17^ is catalytically essential in ICH, where it is proposed to act as a general acid/base (Fig. 1A) (*39*). In wild-type ICH, Asp^17^ is protonated (i.e., a carboxylic acid) in the free enzyme as determined using x-ray bond length analysis (*39*), but the 1.3-Å resolution of the XFEL G150T data is too low for this type of analysis. Therefore, the protonation status of Asp^17^ in the thioimidate intermediate–containing enzyme is unknown. Proton donation by Asp^17^ to the C1 isocyanide-derived carbon atom to form the thioimidate intermediate is proposed to be a key event in catalysis (Fig. 1A) and should change active site electrostatics, which may be important for the catalysis-activated ICH conformational dynamics that we observe. To investigate the influence of Asp^17^ ionization state on solvent structure and conformational heterogeneity in G150T ICH, we performed molecular dynamics (MD) simulations of several unit cell volumes containing the G150T ICH thioimidate intermediate with either a protonated Asp^17^ (residue name ASH, corresponding to the carboxylic acid) or a deprotonated Asp^17^ (residue name ASP, corresponding to the carboxylate) (Methods). The method of performing MD simulations of volumes of the crystal lattice that we use here is closely related to those applied to study solvent structure (*63*–*65*) and conformational variability (*65*).

**Fig. 6.**
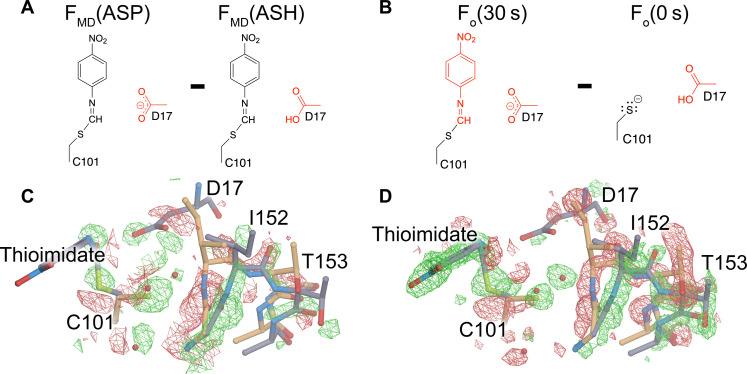
Deprotonation of Asp^17^ causes major conformational changes in the active site. (**A**) Differing protonation states of Asp^17^ that were used to calculate structure factors (*F*_MD_) in crystalline MD simulations. (**B**) Corresponding state of the enzyme active site in the experimental *F*_o_(30 s)-*F*_o_(0 s) isomorphous difference electron density map. (**C**) Calculated isomorphous difference electron density map described in (A) contoured at 6σ. (**D**) Experimental difference electron density map for the active site contoured at 3σ. The excellent agreement between these maps (C and D) demonstrates that water entry and changes in the active site conformational ensemble upon thioimidate formation are driven by deprotonation of Asp^17^.

To analyze the simulations in the context of the experimental data, we use the MD-MX procedure (*65*). In the MD-MX procedure, structure factors and electron density maps are calculated from the ensemble that was sampled in the simulated crystal volume during the MD trajectory (*65*). This computational time and lattice averaging is similar to what occurs during x-ray diffraction from a crystal and thus facilitates the direct comparison of the simulation to experiment. We used these simulation trajectory–derived structure factors (called *F*_MD_ hereafter) to calculate isomorphous difference electron density maps by subtracting the MD structure factors for G150T ICH-thioimidate with a protonated Asp^17^ (ASH) from the same form of the protein with a deprotonated Asp^17^ [i.e., *F*_MD_(ASP)-*F*_MD_(ASH)] (Fig. 6A). The MD-MX difference map indicates that Asp^17^ deprotonation reorganizes solvent in the active site and redistributes the populations of the alternate conformers in the I152 loop (Fig. 6C). Because the Cys^101^-thioimidate is present in both simulations, Asp^17^ deprotonation alone is responsible for this change in active site dynamics. Comparison of the *F*_MD_(ASP)-*F*_MD_(ASH) difference electron density map (Fig. 6C) to the experimental *F*_o_(30 s)-*F*_o_(0 s) difference map (Fig. 6, B and D) shows a remarkable agreement in features in the active site, demonstrating that deprotonation of Asp^17^ explains the additional correlated loop displacement and solvent entry observed in the G150T ICH XFEL experiment. The correlation coefficient between the *F*_MD_(ASP)-*F*_MD_(ASH) and *F*_o_(30 s)-*F*_o_(0 s) difference electron density maps for main-chain atoms in residues 151 to 154 is 0.58. By contrast, the main-chain map correlation coefficient over the whole protein is 0.08, which is low because the experimental *F*_o_(30 s)-*F*_o_(0 s) map reports on all changes due to thioimidate formation, while the *F*_MD_(ASP)-*F*_MD_(ASH) map reports only on those changes arising from Asp^17^ ionization. Therefore, MD-MX analysis supports the mechanism in Fig. 1A and demonstrates that conformational heterogeneity in the ICH active site is highly sensitive to the protonation state of Asp^17^, which changes during catalysis. In combination with the previously demonstrated importance of Cys^101^ ionization for the initial helix H displacement (*33*), these results underscore the tight coupling between active site electrostatics and enzyme dynamics in ICH.

## DISCUSSION

**Fig. 7.**
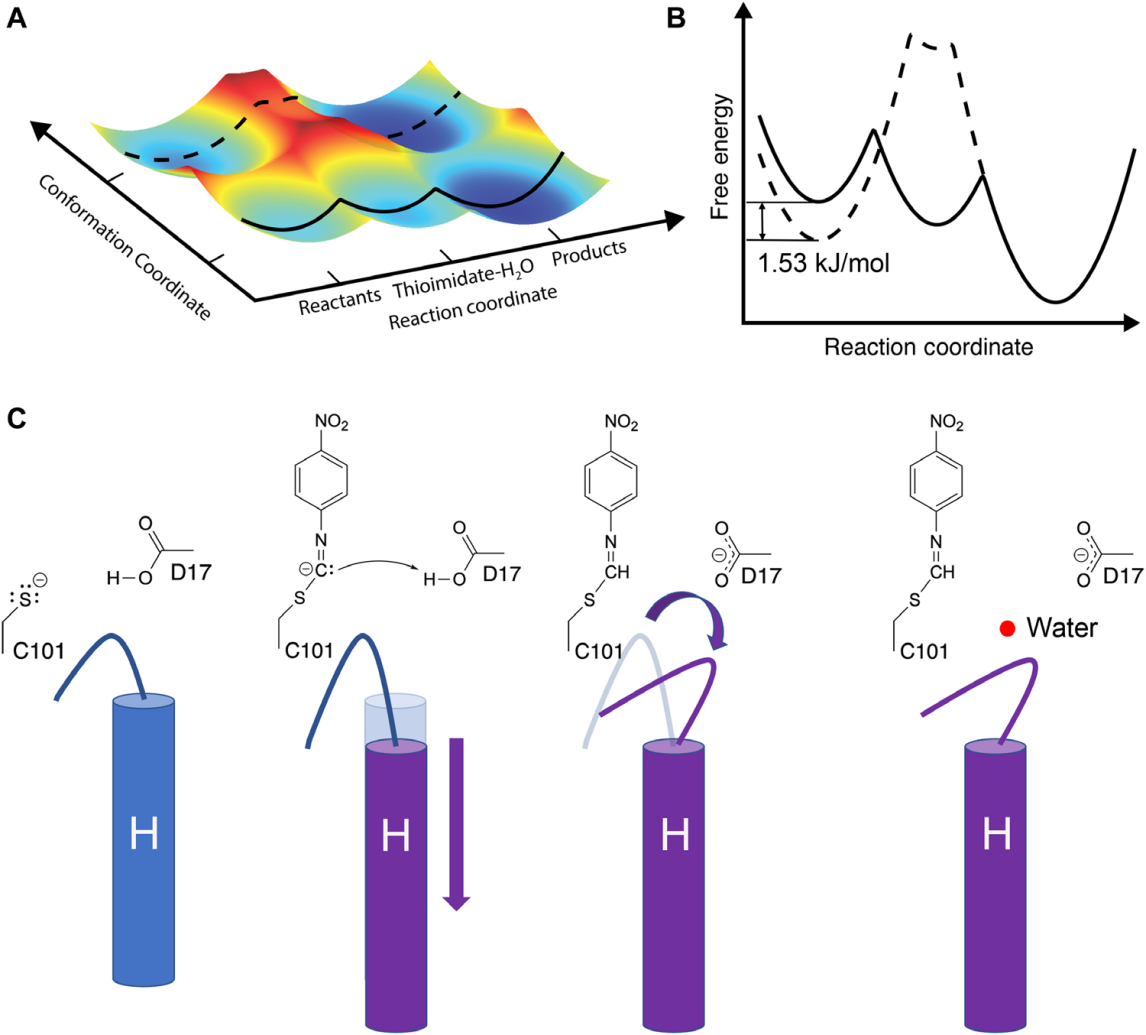
Energy landscape model of ICH catalysis and charge-coupled enzyme dynamics. (**A**) Plot of free energy of the two active site loop conformations versus progress along the reaction coordinate as inferred from XFEL structures. As the reaction proceeds through the intermediate, the open-loop conformation (bottom, with solid line) is energetically preferred. (**B**) Slices through the surface are show in a conventional two-dimensional free energy versus reaction coordinate plot. The 1.53 kJ/mol energy difference of the loop conformations in the resting enzyme is calculated from the crystallographic occupancies using the Gibbs free energy equation (see Methods). The closed-loop conformation (dotted line) has a higher energy than the open one upon intermediate formation and concomitant Asp^17^ ionization. (**C**) Schematic of the first steps in ICH catalysis. Neutralization of charge on Cys^101^ triggers an initial shift in helix H, followed by ionization of Asp^17^, causing a change in loop position that allows water (red sphere) to enter the active site and hydrolyze the intermediate.

MISC allows crystalline enzymes to be observed during catalysis, opening a window into nonequilibrium conformational changes that have been difficult to observe using other approaches. The 1.3-Å resolution of these data is unusually high for room temperature XFEL serial crystallography and permits a detailed modeling of conformational disorder as well as the refinement of anisotropic ADPs. We find that introduction of the substrate selects catalytically competent conformations of the G150T ICH active site and that there are pathways of correlated atomic motion that extend across the enzyme dimer. Interpreting our results from an energy landscape perspective (*4*), binding of substrate to the G150T active site changes the depths of preexisting energy minima, thereby altering populations of the conformers in the ensemble in the absence of large overall structural changes and favoring a subset of possible trajectories along the energy surface (Fig. 7, A and B). We note that it is difficult to model conformations with populations below approximately 5 to 10% occupancy in x-ray crystallography, so it is possible that some conformations are sampled but insufficiently occupied to be modeled. Bearing this limitation in mind, the resting G150T ensemble contains the major conformations observed upon formation of the thioimidate intermediate, while wild-type ICH populates distinct conformations during catalysis in response to covalent modification of Cys^101^ (*33*). Because the G150T mutation favors conformations that are sampled at low occupancy by the wild-type enzyme during catalysis, it provides an example of the value of using mutagenesis to increase the populations of regions of conformational space that are difficult to observe in wild-type enzymes.

Previous pre–steady-state kinetic results indicate that the G150T mutation slows steps both before and after thioimidate formation (*33*). The slowness of G150T was an asset for this study, as it allowed for long substrate mixing times (30 s in our case) and accumulation of the thioimidate intermediate and active site water molecule. It is tempting to ascribe the catalytic impairment of G150T to the conformational disorder we observe at Cys^101^. However, the ~0.2 refined occupancy of the catalytically incompetent Cys^101^ rotamer does not explain the ~80% decrease in *k*_cat_ (steady state) or the ~60% decrease in the pre–steady-state burst rate constant for G150T ICH compared to the wild-type enzyme (*33*), indicating that there are other contributors to this mutant’s catalytic impairment. In our view, the lost H-bond interaction between the Cys^101^ thiolate and the backbone amide of Ile^152^ is likely to be an important contributor to G150T ICH’s slower kinetics, as this H-bond should affect the Cys^101^ p*K*_a_ value, may indirectly influence the extent of Asp^17^ protonation, and may potentially stabilize the thiolate leaving group during hydrolysis of the thioimidate intermediate.

Despite having nearly identical overall structures, isomorphous difference (*F*_o_-*F*_o_) electron density maps calculated between the 274 K synchrotron and 298 K XFEL datasets for G150T ICH showed features that aligned with principal axes of anisotropic ADPs and with minor shifts in atomic position between these higher temperature structures and a cryogenic (100 K) structure. The overlap between these *F*_o_-*F*_o_ electron density peaks and modes of intrinsic mobility in the protein leads us to propose that the difference in temperature is most likely responsible for these changes. Recognizing that many aspects differ between single-crystal synchrotron and serial XFEL data collection, we cannot definitively determine the cause of these *F*_o_-*F*_o_ electron density features. However, our attribution of these map features to temperature-induced changes is supported by previous work using multiple temperature and temperature-jump crystallography, which concluded that proteins respond to modest changes in temperature with complex, distributed changes in their conformational ensembles (*28*, *56*). The sensitivity of isomorphous difference maps to moderate changes in temperature (24°C in this case) should be borne in mind when interpreting time-resolved crystallography studies where substantial temperature changes are possible, such as heating during photoinitiation using high-intensity lasers, evaporative cooling during liquid jet sample delivery in vacuum, or mixing liquids of differing temperatures.

The entry of a water into the G150T ICH active site upon thioimidate formation is intriguing, as it offers a view of an important step before intermediate hydrolysis. Although we cannot be certain that this water is the hydrolytic water, we observe this water entering the active site near the thioimidate intermediate only during catalysis and thus it is likely to be functionally important. Movement of the I152 loop in the shifted helix H conformation is required for water to bind at this location, explaining why this water molecule was not observed in the wild-type enzyme-thioimidate structure, where the helix is predominantly in the unshifted conformation (*33*). In contrast, helix H is constitutively shifted in G150T ICH-thioimidate, and therefore, this rare event in the wild-type enzyme is sampled enough to permit modeling in G150T ICH. Comparison of experimental and MD-MX electron density maps shows that the deprotonation of Asp^17^ is a major driver of the additional loop motion and water binding in the active site upon thioimidate formation. The MD-MX result explains why the crystal structures of wild-type ICH at low pH do not show electron density supporting either the additional 152 loop motion or the water. Although these wild-type ICH structures have a shifted helix H due to Cys^101^ protonation, Asp^17^ is also protonated. By contrast, the postulated charge state of the active site containing the thioimidate intermediate is a neutral sulfur atom and a deprotonated (carboxylate) Asp^17^ (Fig. 1A), which cannot be experimentally recapitulated by manipulating pH alone. These results highlight the value of using MD-MX to study changes in enzyme conformational dynamics that are coupled to active site electrostatics, because directly determining ionization states is extremely difficult using macromolecular x-ray crystallography alone. MD-MX provides a powerful means to manipulate ionization states in silico and then compare the structure factors calculated from these simulations directly to time-resolved experimental data using isomorphous difference electron density maps, as we have done here. The combination of MD simulation and x-ray crystallography experiments suggests a mechanism for how changes in ICH active site electrostatics modulate the enzyme conformational ensemble as a function of catalysis (Fig. 7C). We note that certain mechanistic features in Fig. 7C are not settled, such as whether the substrate is protonated before or after attack by the thiolate, and we have chosen the mechanism that best explains current data.

A challenge in time-resolved crystallography is interpreting electron density maps that result from stochastic conformational changes that are not synchronized among unit cells, leading to a blurring of the electron density and difficulties in its interpretation. The present study demonstrates the value of using mutants to enrich enzyme ensembles in rare conformations that are difficult to study in wild-type enzymes. In combination with experimental interventions that alter enzyme kinetics to favor accumulation of reaction intermediates, mutational probing of conformational ensembles can extend the utility of time-resolved crystallography to more difficult systems or less kinetically accessible regions of their reaction coordinates. Because ICH diffracts to high resolution, has correlated dynamics that change during catalysis, and exemplifies several mechanistic features common to cysteine-dependent enzymes, it is a valuable model system for developing new approaches for time-resolved structural enzymology.

## METHODS

### G150T ICH protein purification, crystal growth, and conventional x-ray data collection

The G150T mutant of *Pseudomonas fluorescens* isocyanide hydratase (PfICH) G150T was purified as previously described (*33*). Briefly, 1.5-liter cultures of LB were inoculated and grown to 0.4 OD_600_ (optical density at 600 nm) at 37°C. Protein expression was induced with 200 μM isopropyl-β-d-thiogalactopyranoside (IPTG) for 4 hours at 37°C, followed by cell harvest by centrifugation, resuspension in lysis buffer [50 mM Hepes (pH 7.5) and 300 mM NaCl], lysis by sonication, and centrifugation to remove cell debris. The supernatant was passed over Ni^2+^-NTA resin (Sigma), and the protein was eluted with 250 mM imidazole (pH 7.0) in lysis buffer. The N-terminal 6×His tag was removed by cleavage with thrombin during dialysis against 25 mM Hepes (pH 7.5), 150 mM KCl at 4°C. Purified protein was then concentrated to 40 mg/ml by centrifugal concentration (Millipore).

G150T ICH crystals used to obtain the cryogenic structure were grown using sitting drop vapor diffusion with protein (23 mg/ml) against a reservoir of 23% polyethylene glycol (PEG) 3350, 200 mM MgCl_2_, 100 mM tris-HCl (pH 8.8), and 2 mM dithiothreitol (DTT) as previously reported (*33*). These crystals were cryoprotected by serial transfer through the reservoir solution supplemented with increasing concentrations of ethylene glycol to a final concentration of 30% (v/v) and then plunge-cooled in liquid nitrogen. The crystal structures of wild-type ICH at various pH values were grown by hanging drop vapor equilibration at 20°C by mixing 2 μl of wild-type ICH protein (20 mg/ml) with 2 μl of reservoir solution. Reservoir solutions contained 23% PEG 3350, 200 mM MgCl_2_, 2 mM DTT, and 100 mM of either tris-HCl (pH 8.3), sodium citrate (pH 6.0), or sodium acetate (pH 4.2, 5.0, or 5.4). Crystals grew in 2 to 7 days and were cryoprotected and plunge-cooled as described above. Crystal growth, mounting, and data collection for the 274 K G150T ICH synchrotron data were previously described (*48*). In that previous study, these data were indexed in an alternative setting in space group I2 for compatibility with a diffuse scattering workflow but were reindexed in space group C2 for this work, permitting calculation of isomorphous difference electron density maps with the XFEL data.

Single-crystal diffraction data were collected at beamlines 12-2 and 9-2 at the Stanford Synchrotron Radiation Lightsource (SSRL) using the oscillation method with shutterless data collection and a Dectris Pilatus 6M pixel-array detector. The wild-type ICH pH series and the cryogenic crystal structure of G150T ICH were collected at 100 K in nylon loops, while the triplicate room temperature G150T synchrotron datasets were collected in 10-μm-thick glass number 50 borosilicate glass capillaries (Hampton Research) at 274 K as previously described (*48*). The data were indexed and integrated using XDS (*66*) and scaled using Aimless (*67*). Data statistics are presented in table S1.

### Microcrystal growth, MISC, XFEL data collection, and processing

Microcrystals of G150T ICH were grown by seeding. G150T ICH crystals (100 to 150) measuring ~100 μm × 50 μm × 50 μm were harvested in stabilizing solution (15.5% PEG 3350, 125 mM MgCl_2_, and 100 mM tris-HCl, pH 8.8) and pulverized by vortexing for ≥10 min with approximately 50 0.5-mm stainless steel beads (Next Advance). The pulverized crystal seed stock was decanted and then centrifuged at 84*g* for 1 min, decanted again, and centrifuged a second time at 325*g* for 1 min to remove larger uncrushed crystal fragments and other detritus. A 1:200 dilution of this seed stock in crystal growth solution (31% PEG 3350, 250 mM MgCl_2_, and 125 mM tris-HCl, pH 8.8) was then mixed with an equal volume of purified G150T PfICH (40 mg/ml in 25 mM Hepes, pH 7.5, 100 mM KCl) and gently mixed by inversion. G150T ICH microcrystals of approximate dimensions 30 μm × 10 μm × 10 μm grew within 30 min, and further growth was quenched by adding 0.5 ml of 1.15× stabilizing solution per 1 ml of crystals. Crystals were stored at room temperature and used within 5 days after growth.

XFEL serial crystallography data were collected at the MFX endstation of the LCLS (*68*) during beamtime mfxlx4418. XFEL pulses at 12 keV with ~1 × 10^12^ photons per 40-fs pulse were delivered to the interaction region at 30-Hz repetition. The x-ray spot size at sample was ~3 μm in diameter. Diffraction data were collected on a Rayonix MX340-XFEL CCD (charge-coupled device) detector operated in 4 × 4 binning mode, and hits were analyzed in real time using OnDA (*69*). The powder diffraction pattern from silver(I) behenate was used to estimate the detector position and the location of the beam center. Joint refinement of the crystal models was performed against the detector position for each batch to account for small time-dependent variations in detector position.

The concentric-flow MESH (coMESH) injector (*70*) was used to deliver G150T ICH microcrystals to the XFEL beam at room temperature and under normal atmosphere composition and pressure. The sample was held in a custom stainless steel sample reservoir that was agitated during the experiment to prevent crystal settling. A Shimadzu LD20 high-performance liquid chromatography (HPLC) pump hydraulically actuated a teflon plunger to advance the sample slurry. A 100 μm × 160 μm × 1.5 m fused silica capillary (Molex) connected the reservoir and filters to the coMESH. This capillary continued unobstructed through the center of the microfluidic tee (IDEX-HS) and into a concentric 250 μm × 360 μm × 500 mm capillary. The capillaries were optically aligned to obtain an approximate 0 mm offset for the free enzyme (0 s) dataset and then were recessed and measured externally to achieve the 62-mm recess for the 30-s substrate mixing dataset. The outer annulus of the coMESH flowed the stabilizing solution through the perpendicular branch of the microfluidic tee in the case of the free dataset and flowed saturated (~2 to 3 mM) p-NPIC substrate in stabilizing solution for the 30-s time points. The p-NPIC substrate was synthesized as previously described (*33*). DTT was omitted from all solutions because it reacts with the p-NPIC substrate. The p-NPIC substrate was loaded into a similar stainless steel reservoir as the crystal slurry and attached to 250 μm × 1/16″ × 1 m polyether ether ketone (PEEK) tubing (Zeuss) connected to the side of the coMESH microfluidic tee junction. Like the crystal slurry, the fluid was actuated by a second HPLC pump (Shimadzu) at 3 μl/min. The 1 m of PEEK tubing was interrupted by a stainless steel union (IDEX-HS) that was electrically charged at +3.1 kV (Stanford Research Systems, SRS PS300). This imparted an electrical charge to the wetted fluid and ultimately focused the ensuing meniscus of mixed sample and stabilizing solution toward the interaction point of MFX. The outer liquid focused the fluids ~0.5 mm away from the outer capillary toward the XFEL focus at the MFX endstation. The charged meniscus was approximately 5 mm away from a grounded counter electrode to complete the electrokinetic focusing. The time delays were assumed to be sufficiently long as compared to the electrokinetic mixing phenomena and were determined by the time the bulk fluid would traverse the offset distance. The flow rates and voltages were held constant during each time point. The flow of the crystals through the main sample capillary (0.1 × 0.16 × 1500 mm) was optically monitored with a 50× objective to assure minimal flow deviations, in addition to the backing pressure of the pumps driving the flows. The stabilizing solution was not introduced to the crystal-carrying slurry until the samples were 62 mm away from the x-ray interaction point. This constraint, coupled with combined volumetric flow rates of 6 μl/min (3 μl/min for the crystal slurry + 3 μl/min for the stabilizing solution with or without substrate), dictated the delay times. Approximately 500 μl of microcrystalline sample was used to measure each time point.

Serial crystallography datasets were reduced and processed using cctbx.xfel and DIALS (*71*, *72*). We indexed 19,135 crystal hits for the free enzyme data and 17,590 crystal hits for the 30-s delay (thioimidate) data. Including lattices from hits with multiple crystals increased the indexing rate (<12.6% of total for free enzyme data and 19.3% for 30-s delay) and these lattices were included in the final postrefinement steps. To increase the indexing rate, we used “subsampling,” where the bright spots found by the DIALS spotfinder on images that failed to index were randomly subsampled to attempt re-indexing. Since it is unknown which reflections from multiple lattices, detector artifacts, or solvent impurities can cause indexing to fail, this procedure removes reflections at random in hopes of finding a subset that will allow indexing. In total, 25 trials are done with each image, randomly subsampling the whole list, in decreasing amounts by 2%, down to 50% of the total, stopping when indexing succeeds. After indexing, time-dependent ensemble refinement was applied, where the data were split up by time and the detector position was refined independently, accounting for experimental drift, as previously described (*71*). Data were scaled and merged to their final resolution cutoffs using cctbx.xfel.merge with errors determined by an alternate formulation of the Ev11 method (*73*). While the details will be described in a subsequent publication, briefly, the error terms are refined using a maximum likelihood method more robust to outliers. For the free enzyme data, 20,796 lattices had their intensities merged and integrated. For the 30-s thioimidate data, 20,372 lattices were indexed and had their intensities merged and integrated. To correct the intensity measurements and perform an initial merge of the data, postrefinement was carried out using cctbx.xfel.merge, using Protein Data Bank (PDB) ID 6NI4 as a scaling and postrefinement reference. The Lorentzian partiality model used parameters unit_cell.value.relative_length_tolerance = 0.3 and outlier.min_corr = −1. The high-resolution cutoff values for the final datasets were determined by previously established resolution cutoff criteria, such as ~10× multiplicity, the point where the *I*/σ(*I*) values become stable, and where CC_1/2_ values stop decreasing monotonically (*74*), indicating that no useful information is contained in resolution shells beyond that point. The data statistics for the XFEL data are provided in table S2.

### Model refinement and analysis

All models were refined in PHENIX (*75*) using riding hydrogen atoms against a maximum likelihood target based on structure factor intensities. Individual anisotropic ADPs were refined for all models except wild-type ICH at pH 4.2 and 5.0, where the translation-libration-screw (TLS) model was used with automatically determined rigid body boundaries in PHENIX. Stereochemical and ADP weights were optimized. Initial G150T ICH XFEL models were based on PDB 6NI4 (*33*) and were manually adjusted in Coot (*76*) and validated using tools in Coot and MolProbity (*77*). Chloride ions were assigned based on the magnitude of the 2m*F*_o_-D*F*_c_ electron density peaks and the presence of weak anomalous difference electron density in the cryogenic G150T ICH structure. Alternate conformations were manually built and refined with grouped occupancies when a contiguous stretch of residues was modeled in alternate positions. However, the thioimidate intermediate only partially modifies Cys^101^ in conformer A; therefore, these occupancies are unequal and a result of a combination of conformational and chemical heterogeneity. The previously collected 274 K synchrotron G150T ICH data were reindexed in C2 and refined as described above. All refined model statistics are provided in table S3.

The difference in ADPs (B-factors) between the 30-s and 0-s G150T ICH XFEL models were calculated for the main-chain atoms and then averaged for each residue using Baverage in the CCP4 suite (*78*). For residues in alternate conformers, the altloc A conformer (which is typically the most occupied conformer) was used for this calculation. Rosenfield difference matrices (*62*) of main-chain atoms were calculated in ANISOANL in the CCP4 suite (*78*) using 30 bins. The free energy difference between the two 151–154 loop conformations in the free enzyme was calculated using the ratio of their refined occupancies and the Gibbs free energy equation: $∆G=-\mathrm{RT}\text{ln}\left(\frac{n2}{n1}\right)$ , where *R* is the ideal gas constant, *T* is temperature in K (298 K in this experiment), and *n_i_* is the occupancy of the *i*th conformer. All isomorphous difference maps were calculated in PHENIX with no weighting. The pH dependence of helix H conformations in Fig. 3B was fitted using the Henderson-Hasselbalch equation:

$$\frac{\left[{\text{Occ}}_{\text{shifted}}\right]}{\left[{\text{Occ}}_{\text{strained}}\right]}=10^{\left(\text{pH}-{pK}_{a}\right)}$$

### Rate versus pH profile of wild-type ICH

The pH dependence of wild-type ICH catalysis was measured using an acetic acid/sodium phosphate double buffer with pH values in the range of 3.5 to 10.25. Solutions were prepared by adjusting the pH of a 2 mM acetic acid and 2 mM sodium phosphate (monobasic) double buffer with small volumes (0.5 to 1 μl) of 500 mM NaOH. Catalysis was initiated by the addition of 0.5 μM wild-type ICH to the double buffer containing 100 μM p-NPIC substrate and monitored by absorbance at 320 nm for 60 s using a Cary50 spectrophotometer. Formation of the product (para-nitrophenyl formamide) was linear in time for all measurements, and the slope of the best-fit line (*A*_320_/second) at each pH was converted to units of μM product/second using an extinction coefficient of 1.33 × 10^4^ M^−1^ cm^−1^ for the product (*33*). After the assay was complete, the pH of each sample was measured at its working concentration using an Orion PerpHecT ROS pH microelectrode (Thermo Fisher Scientific).

### Crystalline MD simulation

Three different crystalline MD systems were prepared, based on the free enzyme and Cys^101^-thioimidate intermediate G150T ICH crystal structures. In one system, the free enzyme crystal structure was used to construct a 2 × 2 × 2 unit cell model of the protein in the crystalline state. Single conformations of residues that were modeled in alternate conformations were assigned to each protomer using random sampling, ensuring that the populations of alternate conformers in the entire simulated system were consistent with the refined occupancies in the crystal structure. In the other two systems, the thioimidate intermediate crystal structure was used to construct a 2 × 2 × 2 unit cell model of the protein in the crystalline state: In one system, Asp^17^ was modeled as unprotonated (ASP17), whereas in the other system Asp^17^ was modeled as protonated (ASH17). For the free enzyme, Cys^101^ was modeled in the deprotonated (thiolate) form. In both systems, Monte Carlo sampling was used to determine the conformation of multi-conformer residues for each protein. However, for the thioimidate intermediate residue, an additional round of Monte Carlo sampling was used to determine whether Cys^101^ in the conformation consistent with covalent modification would have the adduct appended. This was done because Cys^101^ samples two conformations, only one of which is catalytically active and that conformation (altloc A) is only partially modified to the thioimidate. In all cases, cctbx (*75*) methods were used to read in the crystal structure .pdb files and propagate each protein in the state determined by Monte Carlo sampling to a different position in the supercell system. The systems were prepared, solvated, and neutralized with ions using GROMACS version 2022.4 (*79*) methods (pdb2gmx, solvate, and genion*,* respectively). In each system, additional Cl^−^, K^+^, and Mg^2+^ ions were added to best reproduce the concentrations of the crystal buffer (125 mM MgCl_2_, 67 mM tris-HCl, 12 mM Hepes, 50 mM KCl). The protein volume was calculated with the ProteinVolume Online server from gmlab.bio.rpi.edu (*80*), and the total volume of the 32 proteins (8 unit cells, with 4 proteins in each) was subtracted from the volume of the supercell to arrive at a solvent volume of 1.0379 × 10^6^ Å^3^ or 1.0379 × 10^−21^ liters. This solvent volume required an additional 230 Cl^−^ ions, 78 Mg^2+^ ions, and 74 K^+^ ions, on top of the ions required for neutralization (80 K^+^ ions for the apo and ASP17 system, 112 K^+^ ions for the ASH17 systems). The protein and ions were parameterized with the AMBER14SB (*81*) forcefield, and the waters were parameterized with the TIP3P parameter set. The parameters for the Cys^101^-thioimidate intermediate (CYT) residue were determined using MRP.py (*82*), a software package for parametrization of posttranslationally modified residues, which uses AmberTools (*83*) and Gaussian 16 (*84*) to determine the bonded and nonbonded parameters and partial charges.

Supercell systems were subjected to iterative rounds of solvation and equilibration using gmx solvate and gmx mdrun to bring the systems up to pressure in the constant particle number, volume, and temperature (NVT) ensemble (at 300 K). In contrast to the more common number, pressure, and temperature (NPT) ensemble, in which the side of the simulated box is adjusted to tune the pressure, NVT ensembles are necessary for supercell systems to maintain the correct crystalline symmetry and crystal contacts. Production simulation (100 ns) was performed for all systems, with time steps of 2 fs; neighbor searching was performed every 10 steps; the PME algorithm was used for electrostatic interactions with a cutoff of 1 nm; a reciprocal grid of 96 × 80 × 72 cells was used with fourth-order B-spline interpolation; a single cutoff of 1 nm was used for Van der Waals interactions; temperature coupling was done with the V-rescale algorithm. Harmonic positional restraints, with a restraint constant of 200 kJ^−1^ mol^−1^ nm^−2^, were applied to all heavy atoms in the system, with the initial propagated crystal structure supercell positions as the target. The root mean square deviation (RMSD) from the initial heavy atom positions was used to monitor the relaxation of the system under the restraints to a steady-state ensemble, with all systems arriving at steady state at around 60 ns.

The final 10 ns of simulation was used for analysis using the MD-MX procedure introduced by Wych *et al.* (*65*). The xtraj.py Python script (*85*) in the LUNUS GitHub repository (https://github.com/lanl/lunus) was used to calculate the simulated structure factors consistent with the 10 ns of simulation used for analysis. Using this method, it is possible to compute the structure factors from any component of the system; structure factors were computed for the full system, the protein atoms, the water atoms, and the atoms of each ion (Mg^2+^, Cl^−^, and K^+^), individually. “Protein-first refinement” (*65*) was performed with PHENIX (*75*) using simulated intensities computed from the structure factors calculated from just the protein atoms, the structure factors computed from the full system, and the intensities measured in experiment to produce structural models consistent with both the MD and experimental data. For each system, the proteins in the final frame of the simulation were mapped back onto the ASU with cctbx tools using the unit cell and space group information to serve as a representation of the ensemble present across all the proteins in the crystalline system. Here, the procedure was primarily used to produce isomorphous difference maps using the simulated structure factors. The difference maps were computed with sftools from CCP4 (*78*). The full MD-MX procedure was also used to produce revised crystal structures (*65*) with the following *R* factors: *R*_work_ = 0.1492, *R*_free_ = 0.1885 for the free enzyme; *R*_work_ = 0.1584, *R*_free_ = 0.1897 for ASP17; *R*_work_ = 0.1600, *R*_free_ = 0.1989 for ASH17.
