# Supplementary material for: Changes in an enzyme ensemble during catalysis observed by high-resolution XFEL crystallography
Source: Sci Adv. 2024 Mar 27;10(13):eadk7201. doi: 10.1126/sciadv.adk7201 (PMC10971408; doi:10.1126/sciadv.adk7201)
Supplement: Supplementary file 1 — Figs. S1 to S11 Tables S1 to S3 [file sciadv.adk7201_sm.pdf]

Science Advances
AAAS

Supplementary Materials for

Changes in an enzyme ensemble during catalysis observed by high-resolution XFEL crystallography

Nathan Smith *et al.*

Corresponding author: Mark A. Wilson, mwilson13@unl.edu

Sci. Adv. **10**, eadk7201 (2024)
DOI: 10.1126/sciadv.adk7201

This PDF file includes:

Figs. S1 to S11
Tables S1 to S3

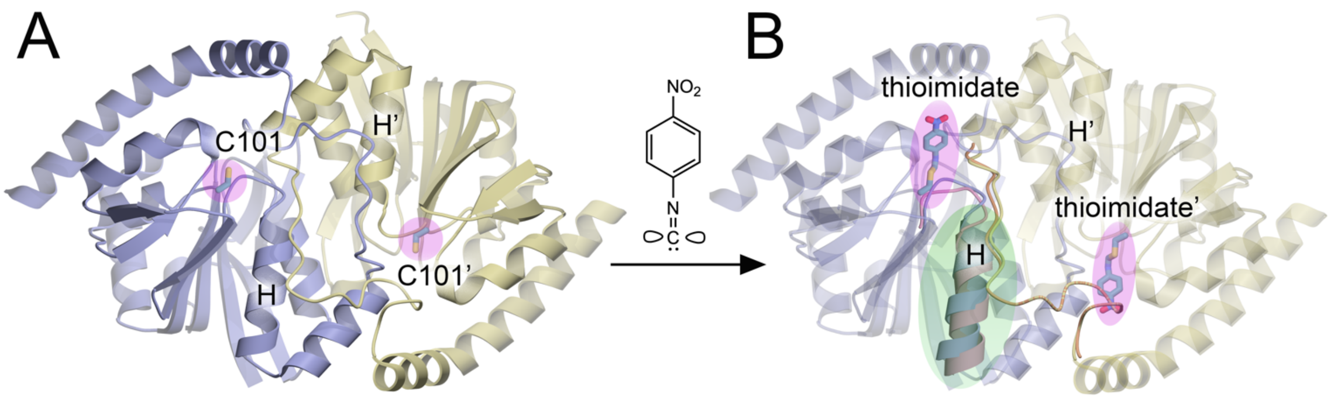

Fig. S1. Schematic of wild-type ICH catalysis-activated motions. (A) Wild-type ICH is shown in ribbon cartoon with the active site highlighted in pink. (B) Upon formation of the thioimidate intermediate, changes in active site hydrogen bonds allow sampling of a shifted conformation of helix H (green highlight).

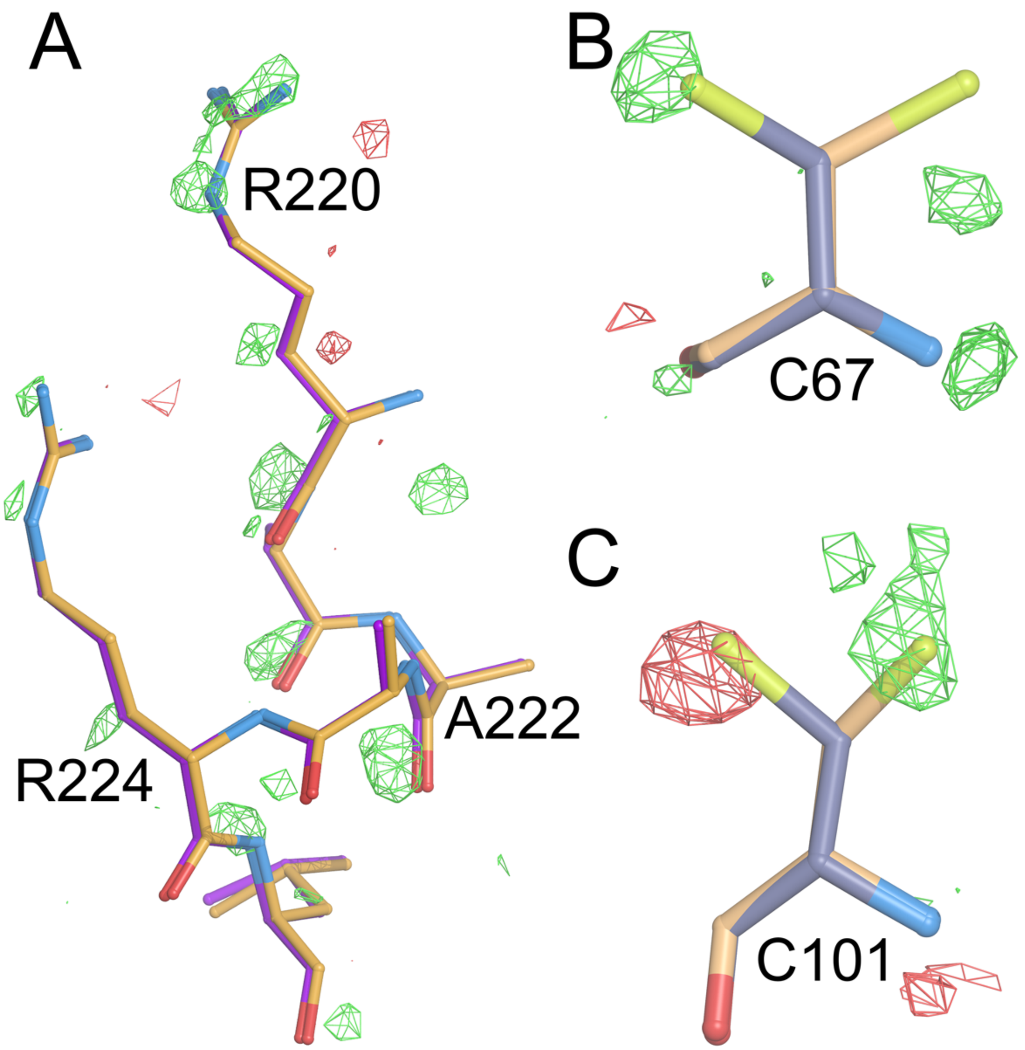

Fig. S2. Examples of temperature-dependent differences in G150T ICH. F_o(274 K synchrotron)-F_o(298 K XFEL) isomorphous difference electron density maps at 1.3 Å resolution are contoured at 3 σ, with positive features in green and negative ones in red. (A) Difference electron density peaks in these residues agree with the small shifts observed when a 100 K cryogenic structure (purple bonds) is superimposed on the 298 K XFEL structure (gold bonds). (B) The populations of C67 alternate conformations are altered by the change in temperature between the synchrotron and XFEL datasets, as shown by difference electron density peaks near the sulfur atom (yellow). (C) The populations of alternate conformations of C101 are altered by change in temperature, as shown by peaks near the sulfur atom (yellow).

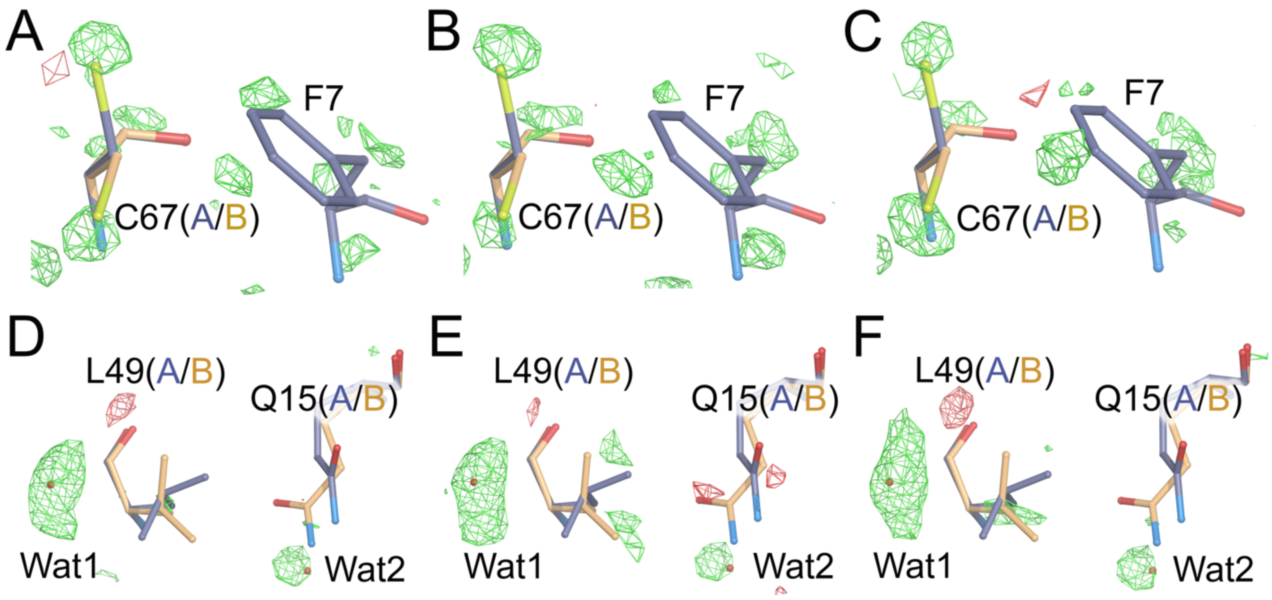

Fig. S3. Difference electron density features between synchrotron and XFEL datasets are reproducible. F_o(274 K synchrotron)-F_o(298 K XFEL) isomorphous difference electron density maps at 1.3 Å resolution are contoured at 2.8-3 σ (corresponding to 0.17-0.18 $e^-/Å^3$) with positive features in green and negative ones in red. (A-C) show the same region near C67 with F_o(274 K synchrotron)-F_o(298 K XFEL) isomorphous difference electron density maps calculated from three independent synchrotron datasets. (D-F) show the region near L49 with F_o(274 K synchrotron)-F_o(298 K XFEL) isomorphous difference electron density maps calculated from three independent synchrotron datasets. The difference electron density features agree closely between the three datasets.

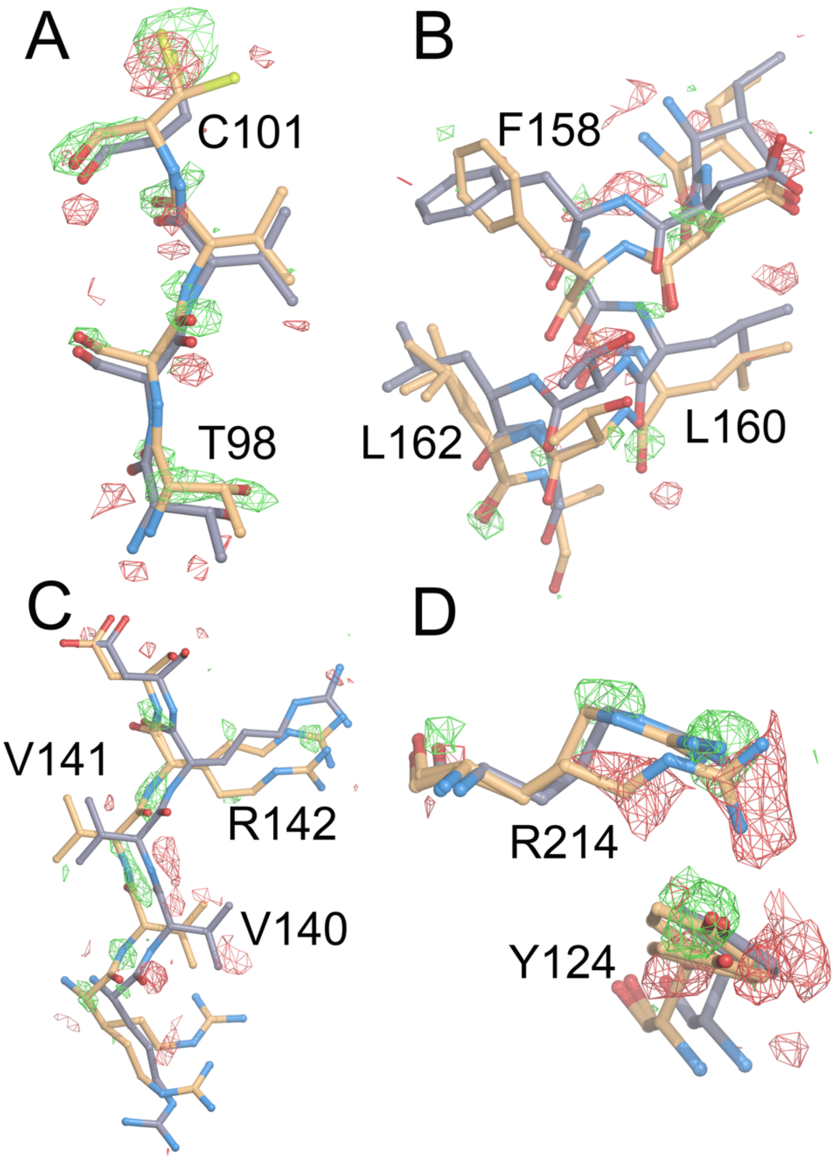

Fig. S4. The G150T mutation causes ICH to populate conformations that the wild-type enzyme samples during catalysis. $F_o(15s)$-$F_o(0s)$ isomorphous difference electron density maps calculated for wild-type ICH from a prior experiment (33) at 1.55 Å resolution are contoured at 2.7σ (corresponding to 0.15 $e^-/Å^3$). Wild-type ICH is shown in slate gray and G150T ICH is shown in gold bonds. (A-D) Selected examples where the conformational shifts evident between the wild-type and G150T ICH structures agree well with the difference electron density map peaks observed during catalysis in wild-type ICH. In all panels, the G150T ICH structure (gold) overlaps with positive difference electron density peaks.

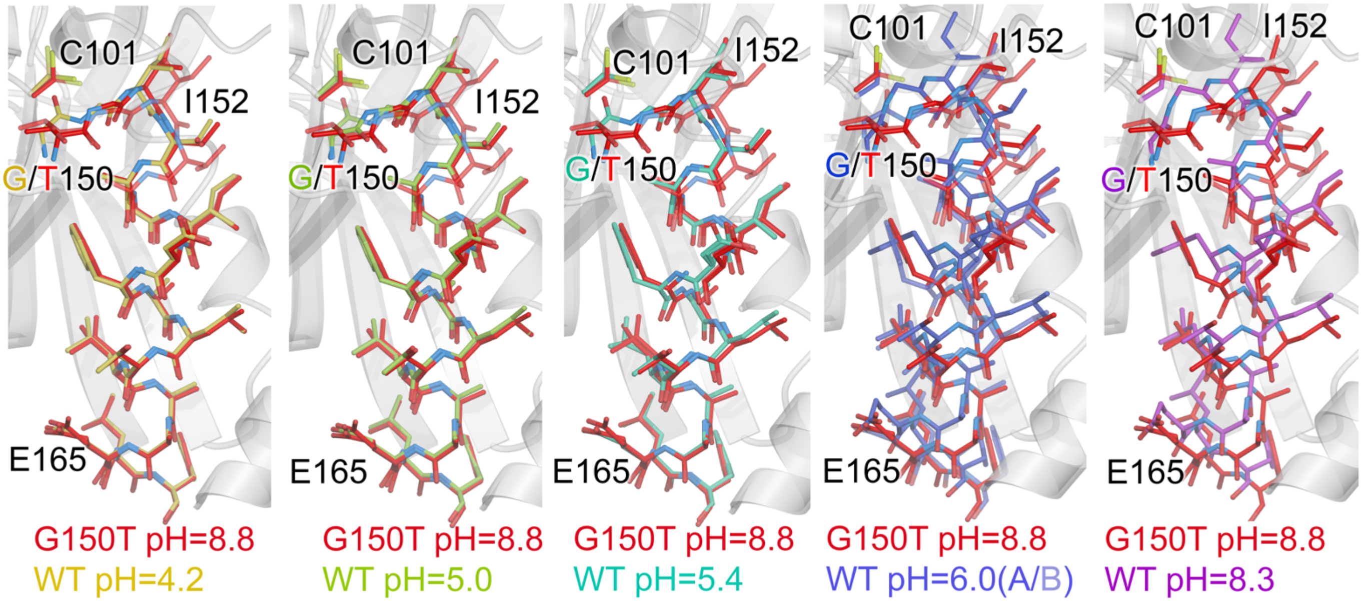

Fig. S5. pH-dependent shifts of the helix H conformation in wild-type ICH recapitulate changes resulting from the G150T mutation. The structure of G150T ICH is shown in red bonds and structures of wild-type (WT) ICH at the indicated pH values are shown in bonds ranging from yellow (pH=4.2) to purple (pH=8.3). As pH decreases, wild-type ICH adopts a conformation that is highly similar to G150T ICH, consistent with changes expected from protonation of C101.

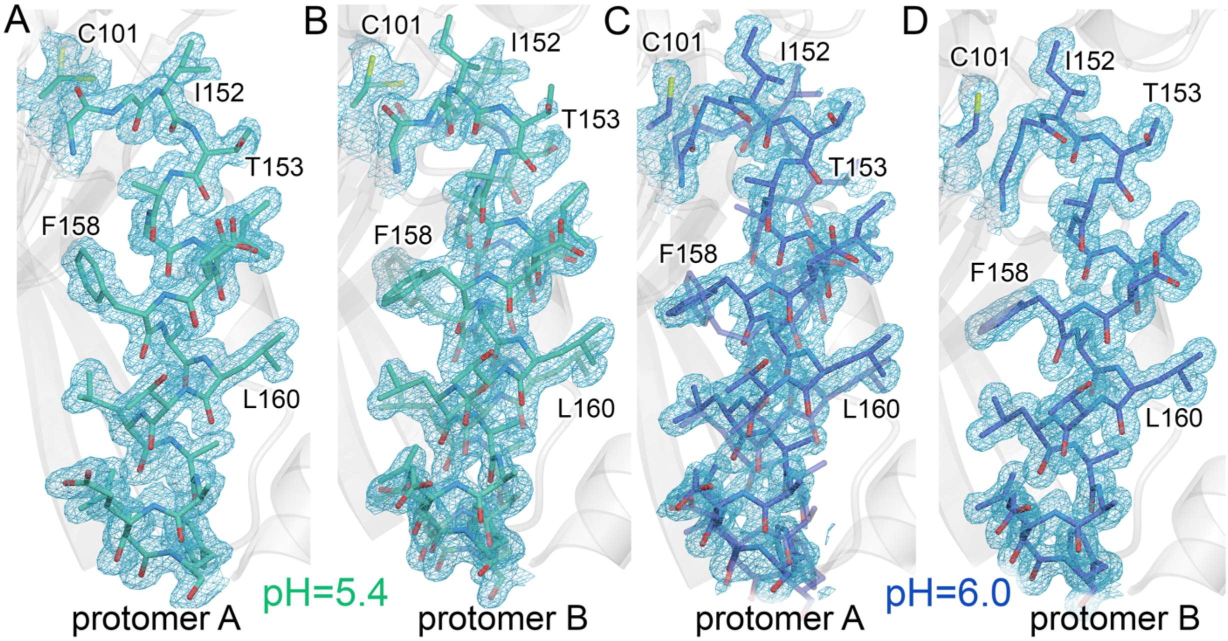

Fig. S6. pH-dependent conformational heterogeneity in helix H is asymmetric in the wild-type ICH dimer. 2mF_o-DF_c electron density is shown contoured at 0.9σ for wild-type ICH at pH=5.4 (1.33 Å resolution, green; A, B) and pH=6.0 (1.2 Å resolution, blue; C, D). The dominant conformer is shown in solid bonds and the minor conformer is semi-transparent. (A,B) At pH=5.4, protomer A is in the fully shifted conformation and protomer B is a mixture of shifted and strained helix conformations. (C,D) At pH=6.0, protomer A is a mixture of shifted and strained helix conformations and protomer B is in the fully strained conformation. In general, the helix in protomer A has a stronger tendency to sample the shifted (G150T-like) conformation.

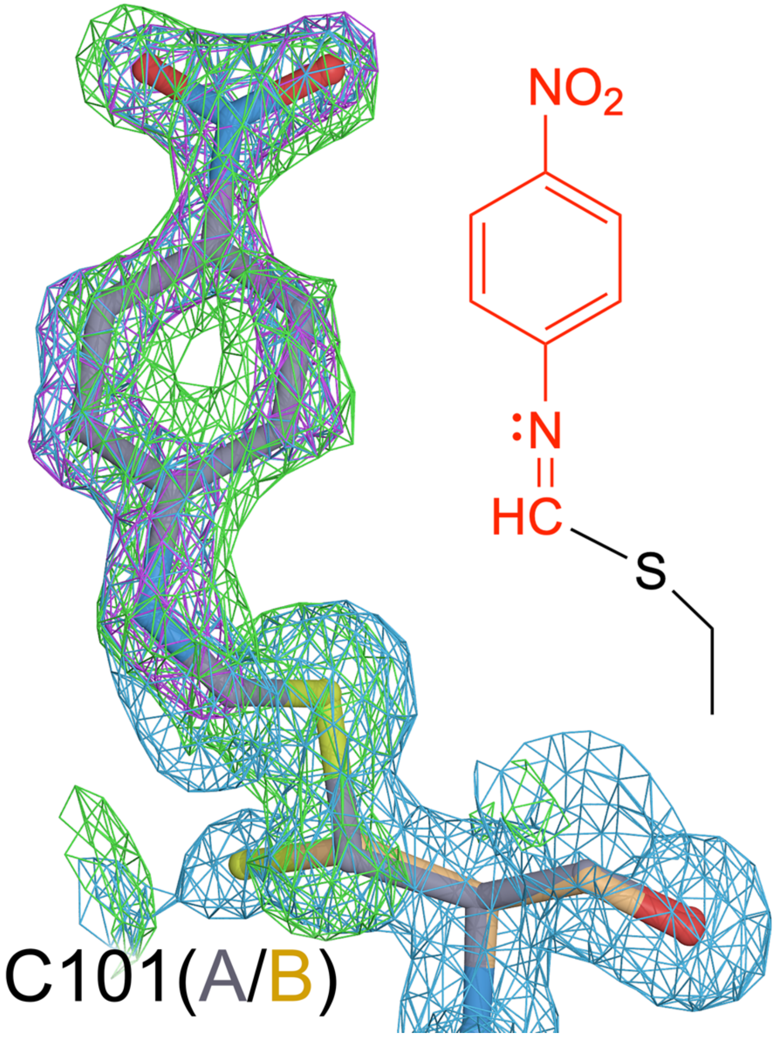

Fig. S7. Thioimidate intermediate forms in G150T ICH 30 seconds after mixing with p-nitrophenyl isocyanide (p-NPIC) substrate. The 1.3 Å resolution F_o(30s)-F_o(0s) isomorphous difference electron density map (2.5σ, green), mF_o-DF_c omit difference electron density map calculated before the model of the p-nitrophenyl thioimidate was included (3.0σ, purple), and the $2mF_o$-DF_c omit electron density map calculated before the model of the thioimidate was included (0.8σ, blue) all show clear evidence for the covalent modification of C101. The chemical structure of the intermediate is shown to the right.

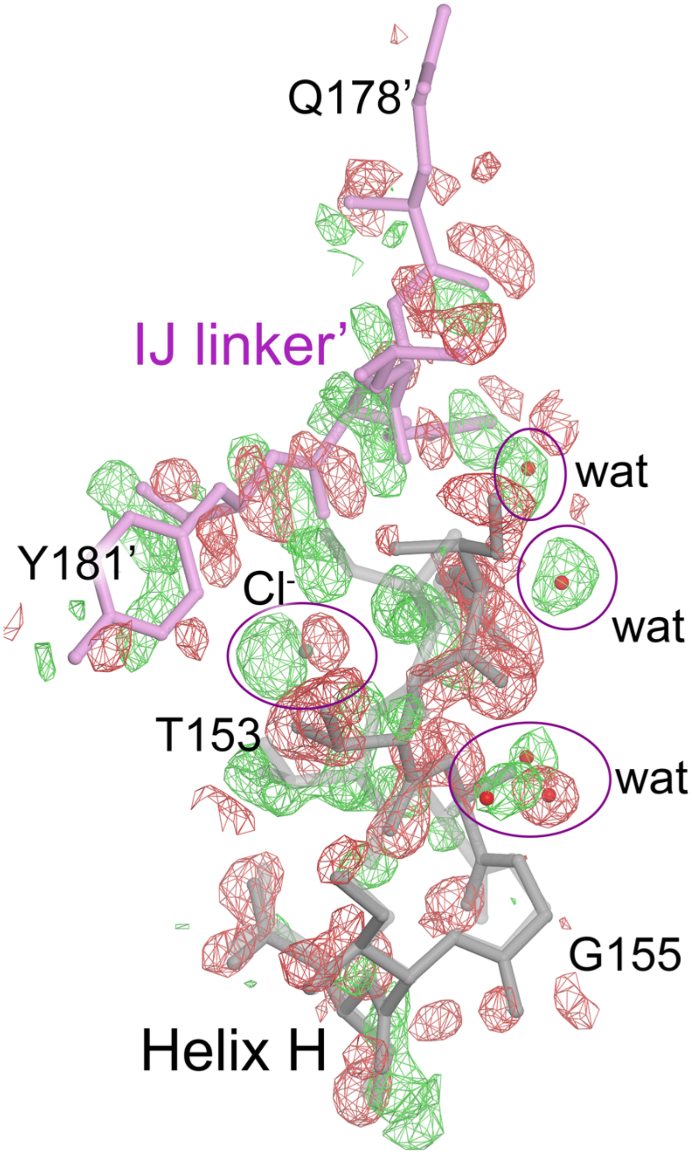

Fig. S8. Correlated changes in protein and solvent during catalysis in G150T ICH. The 1.3 Å resolution $F_o(30s)$-$F_o(0s)$ isomorphous difference electron density map is contoured at 3.0σ with positive features in green and negative ones in red. Helix H, the IJ linker region from the other protomer (purple, indicated with apostrophe), and surrounding solvent (red spheres, labeled “wat”) all display spatially correlated changes in conformation upon intermediate formation during catalysis.

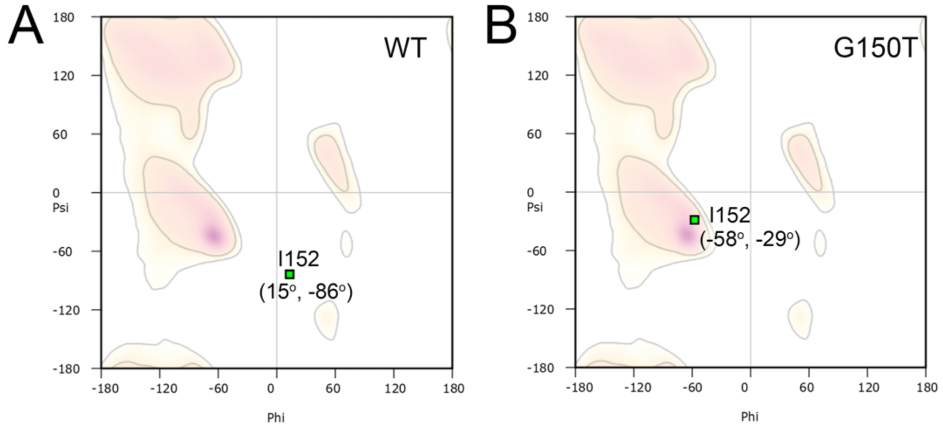

Fig. S9. Ramachandran plot of I152 in wild-type (WT) and G150T ICH. (A) I152 backbone torsions are in a forbidden region of the Ramachandran plot in wild-type ICH (green dot, phi and psi torsion values labeled). This residue is modeled into high-quality electron density and thus the backbone geometry of this residue is not a modelling error. (B) The G150T mutation causes a shift of I152 backbone torsions into the favored region of Ramachandran plot, relieving backbone strain and shifting the position of helix H, which is C-terminal to I152.

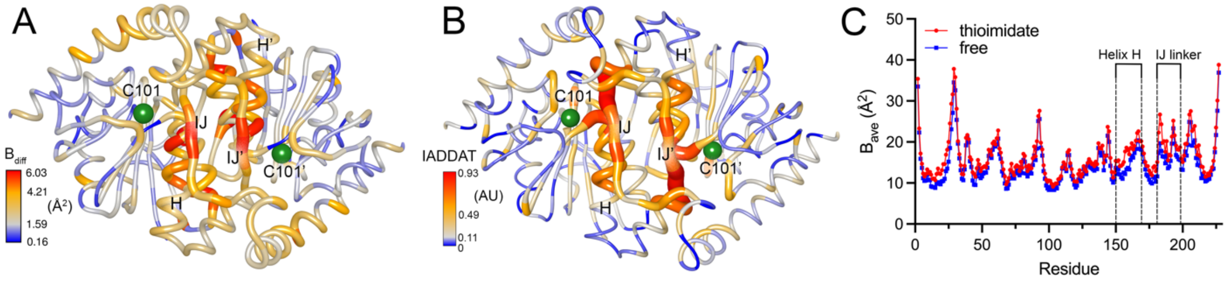

Fig. S10. Isomorphous difference map features during catalysis are localized and overlap with areas of elevated ADPs. (A) Difference ADP values between the thioimidate structure and the free enzyme (B_{diff}=B_{30s}-B_{0s}) are shown, with scale indicated at the lower left. The IJ linker and helix H ADPs are notably elevated upon thioimidate formation. (B) The F_o(30s)-F_o(0s) difference map features were integrated at the absolute difference density above a noise threshold (IADDAT). The threshold was 3σ (corresponding to 0.17 $e^-/Å^3$) within 2.5 Å of the model, which was averaged per residue and mapped onto the dimer. The high IADDAT values in helix H and the IJ linker agree with the pattern of elevated ADPs in (A). (C) Residue-averaged main chain values for the ADPs (B_{ave}) are plotted vs. residue number. Helix H and the IJ linker (labeled) have markedly higher average mainchain ADPs upon thioimidate formation during G150T ICH catalysis compared to the resting free enzyme, as mapped onto the G150T ICH dimer in (A).

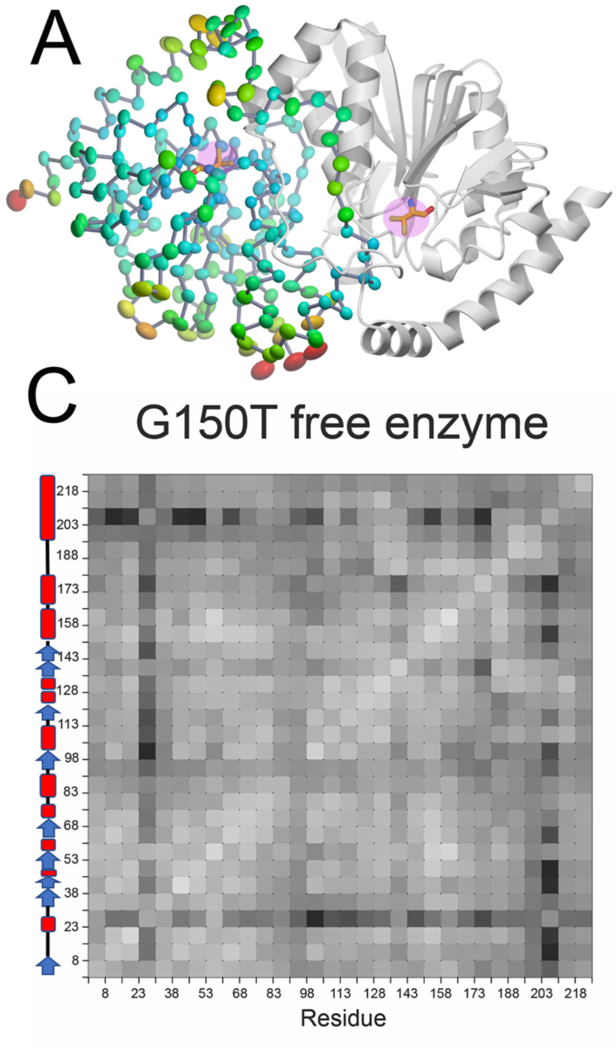

Fig. S11. Anisotropic ADPs in G150T ICH change in magnitude but not direction upon thioimidate formation. (A,B) Anisotropic ADP ellipsoids for Cα atoms at the 85% probability level are colored by magnitude, from blue (5 Å^2) to red (30 Å^2). Formation of the thioimidate intermediate (labeled; pink highlight) during catalysis elevates ADP magnitudes in the active site, the IJ linker, and helix H. Larger ADP magnitudes are indicated with larger ellipsoid volumes and warmer colors. (C,D) Rosenfield difference analysis (62) indicates no major changes in ADP directional preferences upon intermediate formation. Secondary structural elements in ICH are shown on the left to the Rosenfield matrices, with helices as red rectangles and strands as blue arrows. Rosenfield analysis determines the difference between the projections of two atoms' ADP ellipsoids onto the line joining them. Rigid body-like correlated motion of a pair of atoms would result in low difference values (lighter colors). The Rosenfield matrix is computed by averaging mainchain difference ADP projection values in 30 bins (~7 residues per bin).

Table S1. Crystallographic data statistics for synchrotron datasets

Sample	G150T 100 K	G150T-1 274K	G150T-2 274K	G150T-3 274K	WT pH 4.2	WT pH 5.0	WT pH 5.4	WT pH 6.0	WT pH 8.3
Diffraction source	SSRL 12-2	SSRL 12-2	SSRL 12-2	SSRL 12-2	SSRL 9-2	SSRL 9-2	SSRL 9-2	SSRL 9-2	SSRL 9-2
Wavelength (Å)	0.775	0.775	0.775	0.775	0.886	0.886	0.886	0.886	0.886
Temperature (K)	100	274	274	274	100	100	100	100	100
Detector	Pilatus 6M	Pilatus 6M	Pilatus 6M	Pilatus 6M	Pilatus 6M	Pilatus 6M	Pilatus 6M	Pilatus 6M	Pilatus 6M
Space group	$P2_1$	C2	C2	C2	$P2_1$	$P2_1$	$P2_1$	$P2_1$	$P2_1$
a, b, c (Å)	55.56, 58.65, 68.87	72.19, 59.72, 56.30	72.20, 59.73, 56.37	72.13, 59.66, 56.17	57.04, 40.60, 83.28	57.05, 40.72, 83.43	56.53, 56.79, 68.77	56.75, 56.79, 68.41	56.87, 56.83, 68.24
α, β, γ (°)	90.00, 110.92, 90.00	90.00, 115.89, 90.00	90.00, 115.88, 90.00	90.00, 115.86, 90.00	90.00, 104.28, 90.00	90.00, 104.42, 90.00	90.00, 112.44, 90.00	90.00, 112.54, 90.00	90.00, 112.54, 90.00
Mosaicity (°)	0.08	0.10	0.14	0.08	0.12	0.13	0.15	0.10	0.06
Resolution range (Å)	38.87-1.00 (1.02-1.00)	39.47-1.15 (1.17-1.15)	35.21-1.20 (1.22-1.20)	35.16-1.10 (1.12-1.10)	40.35-1.50 (1.53-1.50)	37.34-1.45 (1.47-1.45)	38.45-1.33 (1.36-1.33)	38.52-1.20 (1.22-1.20)	38.57-1.02 (1.04-1.02)
Total no. of observations	1035459 (45149)	280457 (12317)	254082 (11584)	302439 (13882)	323496 (14785)	360375 (16343)	547538 (28370)	725100 (36449)	1238270 (51309)
No. of unique observations	218349 (10505)	74206 (3528)	66132 (3293)	82868 (3983)	58834 (2890)	65010 (3157)	89288 (4619)	123914 (6174)	201545 (10211)
Completeness (%)	98.1(95.6)	97.4 (94.3)	98.3 (95.8)	95.7 (93.4)	98.9 (99.2)	98.4 (97.5)	97.3 (95.8)	99.2 (97.5)	99.4 (98.5)
Multiplicity	4.7 (4.3)	3.8 (3.5)	3.8 (3.5)	3.6 (3.5)	5.5 (5.1)	5.5 (5.2)	6.1 (6.1)	5.9 (5.9)	6.1 (5.0)
⟨I/σ(I)⟩	9.3 (0.5)	12.0 (0.9)	7.3 (1.1)	11.8 (1.0)	9.5 (0.9)	10.1 (1.0)	13.3 (0.8)	9.8 (1.0)	12.5 (0.9)
$CC_{1/2}$	0.999 (0.262)	0.999 (0.366)	0.997 (0.334)	0.998 (0.334)	0.999 (0.404)	0.999 (0.427)	0.999 (0.325)	0.999 (0.435)	0.999 (0.350)
R_{meas}	0.063 (2.850)	0.052 (1.720)	0.078 (2.243)	0.057 (1.839)	0.107 (2.150)	0.097 (1.822)	0.067 (2.504)	0.096 (1.921)	0.071 (1.944)

Table S2. Crystallographic data statistics for XFEL datasets

Sample	G150T apo XFEL	G150T 30s XFEL
Diffraction source	LCLS MFX	LCLS MFX
Wavelength (Å)	1.029	1.029
Temperature (K)	298	298
Detector	Rayonix MX340-XFEL	Rayonix MX340-XFEL
Space group	C2	C2
a, b, c (Å)	72.13, 59.85, 56.14	72.14, 59.76, 56.11
α, β, γ (°)	90.00, 115.89, 90.00	90.00, 115.88, 90.00
Resolution range (Å)	22.00-1.30 (1.32-1.30)	21.98-1.30 (1.32-1.30)
Total number of indexed images	20796	20372
Total no. of observations	2857813 (42449)	2134593 (43514)
No. of unique observations	52766 (2562)	52683 (2580)
Completeness (%)	99.97 (100.0)	99.97 (100.0)
Multiplicity	54.11 (16.43)	40.51 (16.85)
⟨I/σ(I)⟩	5.00 (1.32)	4.70 (1.30)
$CC_{1/2}$	0.986 (0.428)	0.970 (0.527)
R_{split}	0.133 (0.625)	0.163 (0.636)

Table S3. Crystallographic refinement statistics

Sample	G150T XFEL Free	G150T XFEL 30s	G150T cryo	G150T-1 RT	G150T-2 RT	G150T-3 RT	WT ICH pH 4.2	WT ICH pH 5.0	WT ICH pH 5.4	WT ICH pH 6.0	WT ICH pH 8.3
PDB code	8VPW	8VQ1	8TSX	8TSU	8TSY	8TSZ	8TT0	8TT1	8TT2	8TT4	8TT5
Temperature (K)	298	298	100	274	274	274	100	100	100	100	100
Refinement program	PHENIX 1.19.2-4158	PHENIX 1.19.2-4158	PHENIX 1.19.2-4158	PHENIX 1.19.2-4158	PHENIX 1.19.2-4158	PHENIX 1.19.2-4158	PHENIX 1.19.2-4158	PHENIX 1.19.2-4158	PHENIX 1.19.2-4158	PHENIX 1.19.2-4158	PHENIX 1.19.2-4158
Resolution range (Å)	22-1.30 (1.33-1.30)	21.98-1.30 (1.33-1.30)	38.87-1.00 (1.01-1.00)	35.25-1.15 (1.17-1.15)	35.21-1.20 (1.22-1.20)	35.16- 1.10 (1.12-1.10)	40.35-1.50 (1.52-1.50)	32.78-1.45 (1.47-1.45)	38.45-1.33 (1.35-1.33)	38.52-1.20 (1.22-1.20)	38.01-1.02 (1.03-1.02)
Completeness (%)	99.95 (100)	99.98 (100)	95.92 (59)	96.82 (88)	98.13 (95)	95.42 (93)	98.62 (98)	98.07 (96)	96.98 (94)	99.19 (97)	98.69 (82)
No. of reflections	52778 (3609)	52672 (3573)	213316 (4309)	73920 (2347)	66082 (2474)	82841 (2642)	58739 (2606)	64920 (2569)	89186 (2734)	123860 (3786)	200123 (5317)
No. of reflections, test set	1997 (142)	2000 (141)	5143 (101)	3801 (114)	3331 (132)	4122 (144)	2905 (121)	3233 (152)	4336 (138)	6020 (214)	9758 (290)
R_{work}	0.1280 (0.2350)	0.1370 (0.2612)	0.1417 (0.3557)	0.1196 (0.3113)	0.1175 (0.2914)	0.1161 (0.2732)	0.1684 (0.3211)	0.1556 (0.3060)	0.1457 (0.3765)	0.1288 (0.2911)	0.1216 (0.2939)
R_{free}	0.1520 (0.2887)	0.1713 (0.3089)	0.1679 (0.4055)	0.1405 (0.2862)	0.1371 (0.3104)	0.1338 (0.2629)	0.1960 (0.3421)	0.1818 (0.3441)	0.1763 (0.3838)	0.1581 (0.2891)	0.1377 (0.3035)
No. of non-H atoms											
Protein	1987	1963	4116	2002	2002	2002	3642	3567	4280	4518	4180
Water	153	154	447	178	178	181	407	373	433	537	538
Heteroatom	1	12	5	1	1	1	0	0	16	28	28
Total	2141	2129	4568	2181	2181	2184	4049	3940	4729	5083	4746
Average R.M.S. deviations											
Bonds (Å)	0.007	0.005	0.007	0.007	0.010	0.008	0.007	0.005	0.005	0.005	0.008
Angles (°)	0.875	0.787	0.965	0.880	1.029	0.972	0.771	0.746	0.856	0.890	1.020
Average B factors ($\langle B_{iso} \rangle$;Å^2)											
Protein	16.67	18.72	15.34	17.90	18.24	16.57	22.40	21.09	20.89	14.83	12.67
Water	36.54	39.86	32.98	37.05	36.83	33.67	30.43	34.29	37.08	32.15	27.33
Average ADP anisotropy[1]											
Protein	0.498	0.493	0.442	0.430	0.487	0.494	0.561*	0.551*	0.396	0.403	0.458
Water	0.387	0.366	0.394	0.357	0.413	0.404	1.000	0.390*	0.394	0.366	0.394
MolProbity clashscore[2]	2.48	2.25	3.95	1.97	1.72	1.48	1.76	1.11	4.49	2.72	1.76
Ramachandran plot											
Outliers (%)	0.00	0.00	0.00	0.00	0.00	0.00	0.00	0.00	0.00	0.44	0.44
Allowed (%)	0.89	0.89	0.89	0.89	0.89	0.45	0.89	0.88	1.33	1.11	1.11
Favored (%)	99.11	99.11	99.11	99.11	99.11	99.55	99.11	99.12	98.67	98.45	98.45

[1]Anisotropy is defined as the ratio of the smallest to largest eigenvalue of the ADP tensor.
[2]Defined as the number of close contacts (atom overlap > 0.4 Å) per 1000 atoms.
